# What is the carbon footprint of academic clinical trials? A study of hotspots in 10 trials

**DOI:** 10.1136/bmjopen-2024-088600

**Published:** 2024-10-16

**Authors:** Jessica Griffiths, Fiona Adshead, Rustam Al-Shahi Salman, Craig Anderson, Emma Bedson, Judith Bliss, Ana Boshoff, Xiaoying Chen, Denise Cranley, Peter Doran, Fidelma Dunne, Carrol Gamble, Katie Gillies, Kerenza Hood, Columb Kavanagh, Julia Malone, Naomi McGregor, Carolyn McNamara, Elis Midha, Keith Moore, Lucy Murphy, Christine Newman, Seamus O'Reilly, Alexis M Perkins, Sarah Pett, Matthew Robert Sydes, Laura Whitty, Frank You, Lisa Fox, Paula R Williamson

**Affiliations:** 1 Clinical Trials and Statistics Unit, The Institute of Cancer Research, London, UK; 2 Sustainable Healthcare Coalition, London, UK; 3 Edinburgh Clinical Trials Unit, The University of Edinburgh, Edinburgh, UK; 4 George Institute for Global Health, Camperdown, New South Wales, Australia; 5 Liverpool Clinical Trials Centre, University of Liverpool, Liverpool, UK; 6 Imperial Clinical Trials Unit, Imperial College London, London, UK; 7 University College Dublin, Dublin, Ireland; 8 CTN Diabetes and Institute for Clinical Trials, University of Galway, Galway, Ireland; 9 Health Services Research Unit, University of Aberdeen, Aberdeen, UK; 10 Centre for Trials Research, Cardiff University, Cardiff, UK; 11 Trinity College Dublin, Dublin, Ireland; 12 Cancer Trials Ireland, Dublin, Ireland; 13 Newcastle Clinical Trials Unit, Newcastle University, Newcastle upon Tyne, UK; 14 Technology & Innovation, KCR, London, UK; 15 Cardiff University School of Medicine, Cardiff, UK; 16 MRC Clinical Trials Unit, Institute of Clinical Trials and Methodology, University College London, London, UK; 17 Health Data Research UK, London, UK; 18 University of Liverpool, Liverpool, UK

**Keywords:** clinical trial, climate change, statistics & research methods, randomized controlled trial

## Abstract

**Abstract:**

**Background:**

Clinical trials are fundamental to healthcare, however, they also contribute to anthropogenic climate change. Following previous work to develop and test a method and guidance to calculate the carbon footprint of clinical trials, we have now applied the guidance to 10 further UK and international, academically sponsored clinical trials to continue the identification of hotspots and opportunities for lower carbon trial design.

**Methods:**

10 collaborating clinical trial units (CTUs) self-identified and a trial was selected from their portfolio to represent a variety of designs, health areas and interventions. Trial activity data was collated by trial teams across 10 modules spanning trial setup through to closure, then multiplied by emission factors provided in the guidance to calculate the carbon footprint. Feedback was collected from trial teams on the process, experience and ease of use of the guidance.

**Results:**

We footprinted 10 trials: 6 investigational medicinal product trials, 1 nutritional, 1 surgical, 1 health surveillance and one complex intervention trial. Six of these were completed and four ongoing (two in follow-up and two recruiting). The carbon footprint of the 10 trials ranged from 16 to 765 tonnes CO_2_e. Common hotspots were identified as CTU emissions, trial-specific patient assessments and trial team meetings and travel. Hotspots for specific trial designs were also identified. The time taken to collate activity data and complete carbon calculations ranged from 5 to 60 hours. The draft guidance was updated to include new activities identified from the 10 trials and in response to user feedback.

**Discussion:**

There are opportunities to reduce the impact of trials across all modules, particularly trial-specific meetings and travel, patient assessments and laboratory practice. A trial’s carbon footprint should be considered at the design stage, but work is required to make this common place.

**STRENGTHS AND LIMITATIONS OF THIS STUDY**

- The guidance is intended for use by triallists who have no prior experience of carbon footprinting.
- The guidance was applied to a wide variety of trial designs, health areas and interventions.
- The most up-to-date and publicly available emission factors are used in the calculations and where available, country-specific information was used.
- Emission factors may differ from those applicable at the time the trials were conducted, and more up-to-date emission factors may be available, often via paid subscription.
- The guidance is limited to calculation of greenhouse gas emissions and does not currently extend to other environmental impacts such as water and air quality.

## Introduction

 Human health and climate change are inextricably linked; pollution, extreme weather events, poverty, malnutrition and increased disease result in an increased need for healthcare, which in itself is responsible for 4%–5% of global greenhouse gas (GHG) emissions. Clinical trials are a fundamental part of routine health and social care and are critical to the evaluation of new health interventions. Yet, they themselves contribute to healthcare greenhouse gas emissions responsible for anthropogenic climate change: approximately 40 000 new trials were registered globally on ClinicalTrials.gov in 2023, with estimated carbon footprints of ~80 to over 2000 tonnes CO_2_e per trial.^1^,^3^ For context, 80 tonnes CO_2_e is equivalent to the GHG gas emissions from the annual footprint of 6 UK citizens,^4^ 49 return flights from London to New York,^5 6^ 200 000 miles driven by an average petrol car^7^ or the electricity used by 16 homes.^7^

This year, for the first time, the average global temperature exceeded the 1.5° threshold for 12 consecutive months.^8^ Now, more than ever, we must take immediate action to prioritise climate change mitigation. As the first step of a strategy to reduce the carbon footprint of clinical trials and contribute to climate change mitigation, we developed a method and detailed guidance to calculate the carbon footprint of a clinical trial. The first iteration of the guidance (V.0.1) was piloted on two trials managed by the Institute of Cancer Research Clinical Trials and Statistics Unit.^2^ Here, we report results from the application of the guidance and method (V.0.4) to 10 further UK and international, academically sponsored clinical trials. We present how we worked with collaborating clinical trial units (CTUs) to apply the guidance; the range of trial designs and interventions studied; emerging carbon ‘hotspots’ and updates made to the guidance as a result of accumulating data and working with collaborators using the guidance for the first time.

## Method

### Trial selection

Collaborating CTUs were those represented on the Trials Methodology Research Partnership (TMRP)^9^ Executive Committee and via the network of UK Clinical Research Collaboration registered CTUs.^10^ Additional CTUs joined the group following presentation of pilot work at the International Clinical Trials Methodology Conference 2022 and via the TMRP Greener Trials group. Discussions held with collaborating CTUs resulted in identification of one trial within each CTU to footprint. Trials were selected to represent a wide variety of trial designs, health areas, interventions and procedures. The collaborating CTUs and their selected trials are presented in table 1.

**Table 1:** Collaborating CTUs and the selected trials

CTU	Trial name	Link to protocol	Description
Cardiff Centre for Trials Research	The UK stand together trial	ISRCTN - ISRCTN12300853: Stand Together: supporting children’ s social and emotional wellbeingwell-being in schools	A two-arm pragmatic multicentre cluster randomised controlled trial which aims to evaluate the effectiveness and cost-effectiveness of KiVa, a school-based anti-bullying programme, in reducing bullying in schools compared with usual practice. 116 primary schools participated from four areas; North Wales, West Midlands, South East and South West England.
Edinburgh Clinical Trials Unit	RESTART	ISRCTN - ISRCTN71907627: REstart or STop Antithrombotics Randomised Trial	A prospective, open, blinded endpoint, parallel-group randomised clinical trial that compared the effects of starting vs avoiding antiplatelet therapy after ICH. The trial recruited 537 participants at 122 hospitals in the UK.
Imperial Clinical Trials Unit	ON-PACE	ISRCTN - ISRCTN12474100: Improving the experience of physical activity in people with severe lung disease using dietary nitrate supplementation with beetroot juice	On-PACE is a double-blind randomised trial investigating whether taking a nutritional supplement is beneficial for people with the most severe form of chronic obstructive pulmonary disease (COPD). The trial will recruit 102 people with COPD who use oxygen at home to take part in a 3-month long clinical trial.
Liverpool Clinical Trials Centre	HEAL-COVID	ISRCTN - ISRCTN15851697: Helping alleviate the longer-term consequences of COVID-19	HElping Alleviate the Longer-term Consequences of COVID-19 (HEAL-COVID), an adaptive platform trial, aims to evaluate the impact of treatments on longer-term morbidity, mortality, re-hospitalisation, symptom burden and quality of life associated with COVID-19. The trial took place across 109 sites and randomised 1245 participants.
MRC Clinical Trials Unit at UCL	MAVMET	Adding MAraViroc & /or METformin for Hepatic Steatosis in People Living With HIV - Full Text View - ClinicalTrials.gov	A multicentre, 48-week randomised controlled factorial trial of adding maraviroc and/or metformin for hepatic steatosis in HIV-1-infected adults on combination antiretroviral therapy. The trial took place at 6 sites across the UK and recruited 90 participants.
Newcastle Clinical Trials Unit	PREMISE	ISRCTN - ISRCTN50571778: PREMISE: a surgical trial of minimally invasive treatments of prostate obstruction of the bladder	A multi-arm, multicentre, non-inferiority randomised controlled trial comparing 3 minimally invasive treatments to the current gold standard operation for bladder obstruction due to enlarged prostate in the National Health Service. The planned sample size is 536.
The George Institute	INTERACT3	Study Details | The Third, Intensive Care Bundle With Blood Pressure Reduction in Acute Cerebral HemorrhageHaemorrhage Trial | ClinicalTrials.gov	An international, multicentre, prospective, stepped wedge, cluster randomised, blinded outcome assessed, controlled trial of a care bundle of physiological control strategies in acute intracerebral haemorrhage. The trial recruited 7064 patients from 122 hospitals in 10 countries (Chile, Brazil, China, India, Mexico, Nigeria, Pakistan, Peru, Sri Lanka and Vietnam).
University of Galway	EMERGE	Study Details | A RandomizedRandomised Placebo Controlled Trial of the Effectiveness of Metformin in Addition to Usual Care in the Reduction of Gestational Diabetes Mellitus Effects | ClinicalTrials.gov	A randomised placebo-controlled trial of the Effectiveness of MEtformin in addition to usual care in the Reduction of GEstational diabetes mellitus effects. The trial recruited 535 participants to one site in Galway, Ireland.
Cancer Trials Ireland	Shamrock	Study Details | Neoadjuvant Trastuzumab Deruxtecan (T-DXd) With Response-directed Definitive Therapy in Early Stage HER2-positive Breast Cancer (SHAMROCK Study) | ClinicalTrials.gov	An investigator initiated phase II trial of Trastuzumab deruxtecan in the neoadjuvant treatment of patients with early-stage HER-2 positive breast cancer which will recruit 80 patients in 5 centres in the Ireland.
The Centre for Healthcare Randomised Trials	INTERVAL	ISRCTN - ISRCTN95933794: INTERVAL Dental Recalls Trial	A UK multicentre randomised controlled trial evaluating the effectiveness and cost-effectiveness of three dental recall strategies. The trial recruited 2372 participants across 50 dental practices in the UK.

CTUsclinical trial unitsICHintracerebral haemorrhage

### Calculation of trial carbon footprint

The 10 trials were carbon footprinted by members of the trial management team or research staff at the participating CTUs, or JG, using the guidance previously developed.^2^

The approach to the carbon footprint calculations varied for each collaborating CTU depending on resource available to support the activity. For seven trials, activity data was gathered, and the calculations completed, by the trial team, research staff or MSc students embedded within the CTU. Assistance and support were provided to the CTUs by JG and LF via email, video conferencing and document review. Where CTU staff resources were limited in three of the CTUs, JG carbon footprinted the trials using activity data provided by the trial manager or chief investigator via completion of a data collection questionnaire, or through trial protocols and information gathering meetings. The data collection questionnaire was developed by Edinburgh CTU trial manager Denise Cranley to collate the required trial activity data and subsequently adapted into a template by JG and LF which is included in online supplemental appendix A.

As described in our recent publication,^2^ to estimate the carbon footprint of a clinical trial, the trial activities undertaken to answer the research question which are in addition to routine care must first be identified, then the activity data multiplied by standard emission factors. Activity data are collected across 10 modules which are detailed in table 2. The content and structure of the modules reflect the funding, governance and trial management structures of academically funded clinical trials.

**Table 2:** The 10 data collection modules within the guidance

Module	Scope (activities included)
Trial setup	Production and provision of documentation to sites or patients.
CTU emissions	Energy consumption of trial staff working in an office and commuting or working from home for the duration of the trial.
Trial-specific meetings and travel	Teleconferencing, trial staff travel, sustenance and hotel stays for meetings, site visits, audits and conferences.
Treatment intervention	Shipment of intervention from manufacturer to distributor and/or sites/participants, packaging of intervention and destruction of overage. Manufacture of IMP or other intervention is excluded.
Data collection and exchange	Data collection and storage, for example, emails, trial databases, data linkage, questionnaires, Case Report Forms (CRFs).
Trial supplies and equipment	Equipment used by CTU, supplied to sites or to participants specifically for the trial, for example, IT equipment and wearables, laboratory equipment.
Trial-specific patient assessments	Patient travel and hospital staff time required for trial-specific assessments, for example, scans, bloods, bed days. Only activities undertaken to answer the research question that are in addition to routine care are included.
Samples	Sample kit manufacture and shipment from CTU to sites.
Laboratory	Sample analysis/processing, storage at a central laboratory and/or site laboratories.
Trial close out	Archiving of documentation and ambient samples, return of supplies.

CTUclinical trial unitIMPinvestigational medicinal product

Carbon footprints were calculated using the most up-to-date emission factors that were available at the time of the calculations. The main sources of emission factor used were Ecoinvent V.2.2,^11^ GOV.UK GHG conversion factors^12^ and the SHC care pathway carbon calculator.^13^ More up-to-date factors, or forecasted emission factors, may have been available, however, we want to ensure that the guidance developed can always be used without the need for purchasing any licence to obtain those emission factors, which could be a barrier for publicly funded trialists.

Greenhouse gas emissions produced by an activity will vary depending on where they are conducted as a result of differing energy uses and sources in different countries. Where publicly available, country-specific emission factors and benchmark data sources were used to recalculate the modules with the largest contribution to the total footprint, for example, CTU emissions in the international INTERACT3 trial, to produce a more accurate footprint. Where country-specific information was unavailable, UK data was used as a surrogate for example, for commuting and participant travel in the international INTERACT3 trial.

The guidance includes emission factors for working from home, teleconferencing, telephone consultations and remote data collection, and therefore, adaptations made in trials conducted during COVID-19 were accounted for and reflected in the trial carbon footprints.

The carbon footprint calculations were conducted between January and December 2023. Feedback from users on the time taken to perform the calculations was collected.

### Patient and public involvement

Patients and the public were not involved in this stage of the research to test the guidance on 10 further clinical trials. However, now that a method is available, it is critical that patient views on carbon trade-off decisions relating to participation in research are invited and understood. To facilitate the conversation with patients, an animated video describing sustainable research practices and carbon footprinting of clinical trials coproduced with patients, for patients is in production.

## Results

In total, six investigational medicinal product (IMP) trials were footprinted (in breast cancer, gestational diabetes, COVID-19, intracerebral haemorrhage (n=2) and HIV), one nutritional trial (lung disease), one surgical trial (benign prostate enlargement), one health surveillance trial (dental) and one complex intervention trial (behavioural). Six trials were completed at the time of inclusion and four were ongoing (two recruiting, two in follow-up). Seven trials included UK participation only, two trials were run within the Republic of Ireland and one trial was international (participation from 10 countries, regional trial management and a sponsor CTU-based in Australia). Table 3 provides more details of the trial designs.

**Table 3:** Design and carbon footprint of selected trials

Trial	Carbon footprint (tonnes CO_2_e)	Description	Top three hotspots and their % contribution to total footprint									
			Trial setup	CTU emissions	Meetings and travel	Intervention	Data collection	Trial supplies	Patient assessments	Samples	Laboratory	Trial close out
EMERGE	74	Intervention: IMPCountries: 1Sites: 1Participants: 535Start date: 6 June 2017Trial duration: 6 years		127%				317%	225%			
HEAL-COVID	91	Intervention: IMPCountries: 4 (UK)Sites: 109Participants: 1245Start date: 1 January 2021Trial duration: 4 years		217%			179%					33 %
INTERACT-3	765	Intervention: IMPCountries: 10Sites: 122Participants: 7064Start date: 12 December 2017Trial duration: 6 years		171%	37%				28%			
INTERVAL	61	Intervention: SurveillanceCountries: 4 (UK)Sites: 51Participants: 2372Start date: 1 August 2009Trial duration: 5.5 years		318%	146%				222%			
MAVMET	18	Intervention: IMPCountries: 1Sites: 6Participants: 90Start date: 1 March 2017Trial duration: 5 years							139%		315%	220%
ON-PACE	16	Intervention: NutritionalCountries: 1Sites: 1Participants: 102Start date: 1 June 2022Trial duration: 2.5 years		231%					316%		136%	
PREMISE	25	Intervention: SurgicalCounties: 3 (UK)Sites: 10Participants: 536Start date: 1 April 2022Trial duration: 5 years		154%	311%				227%			
ReSTART	109	Intervention: IMPCountries: 4 (UK)Sites: 122Participants: 537Start date: 1 April 2013Trial duration: 8 years		172%				36%	211%			
SHAMROCK	58	Intervention: IMPCountries: 1Sites: 5Participants: 80Start date: 26 October 2023Trial duration: 7 years		312%					141%		231%	
UK Stand Together	107	Intervention: Complex (behavioural)Countries: 2 (UK)Sites: 116Participants: 12 580Start date: 1 July 2019Trial duration: 2.75 years		235%	314%			149%				

CTUClinical Trial UnitIMPinvestigational medicinal product

Our initial guidance and method included the majority of clinical trial activities and corresponding emission factors required to calculate the carbon footprint of the 10 selected trials. Where new activities were identified, emission factors were sourced from publications, Life Cycle Analysis databases and articles, and all new activity data and emission factors have been added to the Guidance to create V.0.5. All sources are cited and referenced in the guidance.

**Figure 1:**
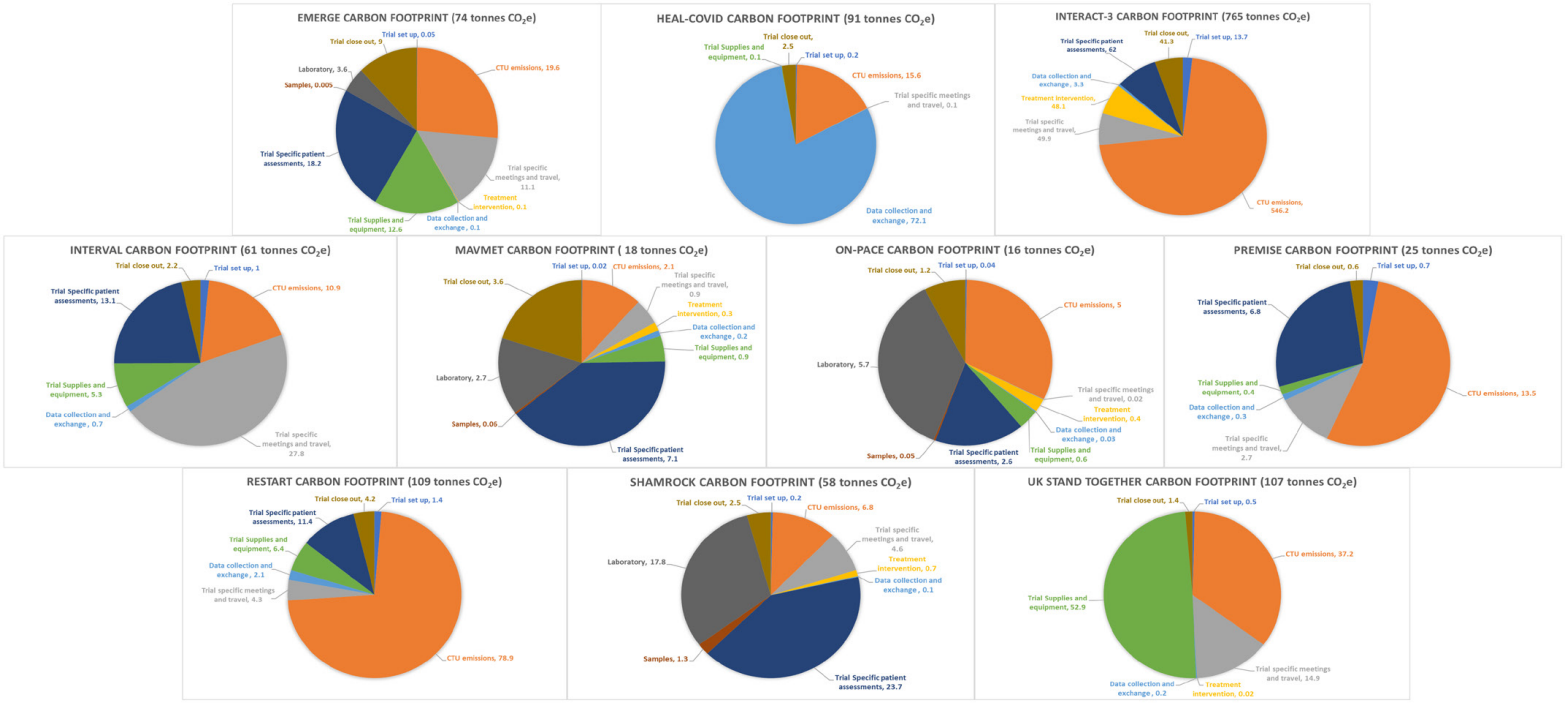
Proportion of greenhouse gas emissions per module in each of the 10 selected trials.

The results of carbon footprinting are presented in table 3, including the total carbon footprint (tonnes CO_2_e) and the three modules which had the largest contributions to the footprint. Figure 1 demonstrates the proportion of greenhouse gas emissions attributed to each module in the 10 trials.

### Total carbon footprint

The estimated trial carbon footprints ranged from 16 tonnes CO_2_e in a single-site study with 102 participants, to 765 tonnes CO_2_e in an international trial which recruited 7064 participants from 122 sites across 10 countries.

### Carbon hotspots

In 9 of the 10 trials, CTU emissions featured in the top 3 hotspots. Typically, this becomes more of a hotspot as the CTU staff full-time equivalent (FTE) increases with an increased number of sites and participants and in trials with a long duration. Contribution from commuting was likely higher prepandemic when most CTU staff were 100% office based. Some of the trials conducted during COVID-19 (MAVMET and HEAL-COVID) also had lower commuting emissions due to staff working from home 100% of their time during lockdowns. The CTU location can also affect commuting emissions; the carbon footprint of commuting was much lower in the CTUs located in London (Imperial and UCL) where public transport is used more in comparison to Edinburgh CTU where over 70% of commuting was by car. CTU emissions also significantly contribute to the carbon footprint of large international trials such as INTERACT-3 due to there being multiple trial coordination centres in multiple countries, some of which have higher intensity national grids than the UK.

In 8 of the 10 trials, trial-specific patient assessments were a hotspot. Patient travel to hospital for visits that were in addition to standard-of-care was frequently a large contributor to this. The absolute contribution in terms of carbon emissions could depend on the location/spread of the trial participant population and the mode of transport generally used. For example, in MAVMET, which was based in London, public transport use was assumed compared with the SHAMROCK trial in Ireland where 100% of participants were assumed to travel by car over larger distances; although trial-specific patient assessments were a hotspot in both trials which were similar in terms of the number of sites and participants, the total carbon emissions was much higher in SHAMROCK.

Staff meetings and travel was a hotspot in four of the trials. This was the largest contributor to emissions in the INTERVAL trial due to travel for site initiation visits, regional recruitment events, monitoring at a portion of the sites, in-person trial management group and trial steering committee meetings and conferences.

Laboratory activity was a hotspot in three of the trials. This is mostly attributed to international shipment of samples/sample kits, or storage of samples in ultra-low temperature freezers, sometimes for up to 10 years.

Trial supplies and equipment were also a hotspot in three of the trials. In the UK Stand Together trial, trial supplies and equipment had the largest contribution to the trial carbon footprint due to provision of 360 tablets to sites for completion of questionnaires. Similarly, in EMERGE, this hotspot was attributed to provision and use of 535 glucometers, and in RESTART, this was related to purchase of IT equipment.

Trial close-out was a hotspot in two of the trials. In MAVMET, this was attributed to storage of 28 archive boxes for 25 years, whereas in HEAL-COVID (in which only 6 of the 10 footprinting modules were relevant as there was a standard of care, locally prescribed intervention and no samples or patient assessments in addition to standard of care), this was attributed to data storage.

In addition to the more general and frequently seen hotspots, these results also illustrated trials with hotspots that were specific to the trial design or intervention.

For example, in HEAL-COVID, data collection and exchange had the largest contribution to emissions due to the considerable cost attributed to accessing and linking data from NHS England (formerly NHSDigital) and purchase of software to operationalise the decentralised trial design. However, in the absence of sufficient activity data and published emission factors, emissions attributed to these activities were calculated using a spend-based emission factor, which are known to be less accurate than activity-based emission factors.^14^ A spend-based approach involves multiplying the cost of an activity or service by an emission factor representing the average emissions per pound spent in that particular industry.

### Feedback on application, use and experience of carbon footprinting guidance

The time reported to collate trial activity data and complete the carbon footprinting calculations ranged from 5 hours to 60 hours, largely depending on trial size and complexity and the extent to which the individual performing the footprinting was familiar with the trial and could easily locate the required information. Collaborators who went on to footprint more than one trial anecdotally noted that it took approximately 50% less time on repeat application of guidance.

Previously, our guidance was applied retrospectively to two completed trials, and we anticipated that application to trials which are currently active or in development would take less time and be less resource-intensive.^2^ To assess this, both ongoing and completed trials were footprinted. In four of the completed trials footprinted by trial teams, the time required to retrospectively collate the trial data alone ranged from 10 to 25 hours, whereas the information was much more readily available in the ongoing trials. For SHAMROCK, the trial setup, most of the anticipated activity was gathered via the protocol, email correspondence and a 1-hour meeting. This is because prospective application of the guidance allows the user to use existing assumptions already made to inform an academic funding application, which can speed up activity data collection. For example, the number of planned trial meetings and patient visits can be taken directly from the funding application of a trial in setup, whereas identifying the number of visits or meetings that actually took place in a trial can require review of multiple folders and databases. However, attempting to make more accurate assumptions can also be more time-consuming. For example, instead of counting the number of boxes stored in an office or looking at the gigabyte of storage used by a trial folder, to estimate this for a trial in setup you would first need to identify a trial with similar number of sites and participants and then use that to estimate the activity data.

The majority of users required very little clarification or help to use the guidance and there were few corrections made to calculations by the project team. However, in some instances, calculation of the trial carbon footprint was iterative which helped to establish and inform where guidance was ambiguous and required clarification.

### New emission factors and activities added to guidance

The guidance from our initial publication has been updated during this application phase to include the following new activities involved in the PREMISE, ON-PACE, UK Stand Together and INTERVAL trials: blood pressure monitoring, saline use, oxygen use, business travel by car, commuting where the mode of transport and distance travelled is known, dental examinations, laptop usage and telephony.

Existing emission factors have been updated in line with 2023 data from GOV.UK. Calculations using electricity and natural gas emission factors were updated, along with freight, business travel, building energy benchmarks and other clinical activities, for example, radiotherapy.^15^

Additional assumptions have been included to aid the user with the calculations, for example, the number of samples that can be stored in a freezer, the number of working hours in one FTE, the number of folders that can be stored in 1 m^2^ and the carbon footprint of common sample kit supplies. The updated ‘detailed guidance and method to calculate the carbon footprint of a clinical trial guidance (V.0.5)’ and associated ‘data collation quick guide and worksheet’ are included as onlinesupplemental appendices B  C, respectively.

## Discussion

### Hotspots

The results presented in this study demonstrate that there are hotspots common to many of the 10 trials, particularly CTU emissions, trial-specific patient assessments and trial meetings and travel.

Despite the variation in total footprint, the median carbon footprint (68 tonnes), is in line with the published pilot trial results (72 and 89 tonnes) and the previous study conducted by Lyle *et al* (average 78 tonnes).^1^ In addition, there is consistency with the three activities accounting for the most CO_2_ emissions (trial team commuting, fuel use at study centres which is included here as CTU emissions and trial team-related travel).

Although results are from a small cohort, the difference in footprint between the national and international trials suggests average footprints should not be calculated across both. The carbon footprint of INTERACT-3, an international trial which enrolled 7064 patients to 122 sites across 10 countries, was 765 tonnes CO_2_e. Although application of the methodology was slightly different, this is comparable to the carbon footprints of the international CRASH-1 and CRASH-2 trials, which recruited 10 000 and 20 200 participants and were estimated to emit 925 and 509 tonnes CO_2_e, respectively.^16^ CTU emissions were the biggest hotspot in INTERACT-3, similarly energy use by trial coordination centre was the largest and second largest contributor to emissions in the CRASH-2 and CRASH-1 trials, respectively. Sample sizes tend to be larger in international trials and they also require a country-specific CTU/Sponsor office to be based in each participating country, which increases the CTU emissions hotspot in such trials.

Our findings were also similar to a study published by Mackillop *et al* of three industry-sponsored late-stage cardiovascular, oncology and respiratory international clinical trials which also identified study team facilities, site monitor visits and trial management meetings as hotspots.^3^ However, at 2498 tonnes CO_2_e, 1638 tonnes CO_2_e and 1437 tonnes CO_2_e, respectively, the absolute carbon footprint of the pharmaceutical industry trials was higher than both the national and international publicly funded/investigator-initiated trial results presented. Inclusion of IMP and placebo manufacture in the Mackillop trials is likely a contributing factor for this, however, future work will explore the differences in relation to trial design and conduct, as well as the method of carbon footprinting.

Patient travel or trial-specific patient assessments were not applicable in the CRASH trials where the outcome was death. They were not identified as hotspots in the Mackillop study and participant-related travel was found only to be the fourth largest contributor to emissions in the Lyle study. Conversely, trial-specific patient assessments were identified as the largest and third largest hotspot in the pilot trials and were a hotspot in eight of the trials presented here.

### Opportunities to reduce

There are opportunities within the control and influence of CTUs to make responsible research decisions and consider alternative trial design approaches which reduce the carbon footprint of a trial without impacting data quality, integrity and validity. Although implementing energy-saving measures and moving to renewable energy sources is generally managed at the research institution level, CTUs can contribute to the reduction of emissions by ensuring staff are aware of and comply with any carbon reduction plans, advocating for and incentivising sustainable commuting, for example, lift share and cycle to work schemes and encouraging participation in workplace sustainability initiatives and groups. Hybrid working will also contribute to reduced CTU emissions due to reduced commuting.

Emissions attributed to in-person patient visits which are in addition to routine care should be considered carefully and reduced where appropriate by considering whether trial-specific assessments and procedures could be carried out virtually or at facilities geographically closer to the patient; carefully considering where additional in-person trial visits can be reduced or combined, for example, with routine care; and allowing e-completion of consent or patient questionnaires where possible. Where participant travel is necessary, where appropriate emissions could be reduced by arranging more sustainable modes of transport such as renewable energy-powered electric vehicles. ON-PACE demonstrated this by use of a green taxi company to transport patients to and from hospital visits. It is vital that trial-specific patient outcomes and their assessment are given greater consideration at the design stage. Heterogeneity in what and how outcomes are measured contributes to research waste which in turn increases emissions due to the need for further studies to be able to answer the research question; the inclusion of core outcome sets, reflecting outcomes of critical importance to decision-makers including people with lived experience, can reduce such research waste and thus provide an opportunity to reduce emissions across the sector as a whole.^17^

The carbon footprint of trial staff meetings and travel can be meaningfully reduced by opting for virtual meetings, remote monitoring (where informed by the trial risk assessment), local monitors, reducing overnight stays and considering more sustainable modes of transport, that is, replacing driving with public transport, discouraging short haul flights to destinations in Europe reachable by train and when travel by air is unavoidable, take direct flights and move from business class to economy. This was demonstrated by the NightLife study, which quantified the carbon and financial savings resulting from changes to the study design in response to the COVID-19 pandemic.^18^ In total, 136 tonnes CO_2_e were saved, 61% of which resulted from online reconfiguration of study meetings and site visits, and virtual attendance at national and international conferences. Guidance on reducing the carbon footprint of monitoring activities for academic trials has been developed by the UK CRC CTU Network Monitoring Task and Finish Group.^19^

To reduce emissions attributed to sample collection and analysis, laboratories could be encouraged to work towards environmental accreditation such as LEAF and My Green Lab, consideration should be given to sample collection time points, frequency and shipment conditions, and the duration and conditions of storage. For example, increasing the temperature of ULT freezers from −80 to −70 can reduce energy consumption by up to 30%.^20^ To minimise the environmental impact of trial supplies and equipment, commercial suppliers can be checked for environmental accreditation such as ISO14001, where possible equipment could be loaned or refurbished instead of buying new and disposed of appropriately. To reduce waste and unnecessary shipments to participating sites, IMP and supplies could be shipped only on identification of eligible patients.

All adaptations to trial design to reduce the carbon footprint need to be balanced against patient acceptability so as not to compromise rigour and further contribute to research waste.

### Limitations

A hotspot may be defined differently between studies and across sectors. We have chosen to highlight the three largest contributors to each trial’s carbon footprint, but the contribution from each module can be seen in figure 1. It is conventional for an activity to be defined as material or significant if it contributes to >10% of the total CO_2_e.^21^ If applying this metric to the results presented, 23 of the 27 hotspots included in table 3 would be deemed significant (contributing to >10% of total CO_2_e). However, it is important to consider processes and activities within trialists’ control which may not be deemed a hotspot but which may be amenable to alternative lower carbon processes. For example, trial setup, which accounts for production and provision of trial information to sites and patients, was not identified as a hotspot in any of the 10 trials. However, with the advent of technological advancements such as electronic Trial Master and Investigator Site Files, processes could be amended to use these lower carbon options.

For trials where the guidance was applied retrospectively, the emission factors used for the carbon footprint of the activities may differ from those available at the time the trial was conducted. As a result, the footprint of certain activities may be under or overestimated, however, it is unlikely to have affected the identification of hotspots within a single trial.

Calculating the carbon footprint of international trials is difficult. Country-specific information must be gathered at a variety of levels to calculate the footprint of a single activity. For example, to calculate emissions attributed to CTU, laboratory and hospital staff FTE, the average amount of space used (m^2^), benchmark energy use of the building type and energy sources must be identified for each country. This information is often unavailable, difficult to find or subject to licence. For the international trial included, country-specific information was used where available and UK emission factors applied in its absence. Although UK-based emission factors cannot be used to calculate the absolute carbon footprint of an international trial, they could be used as a starting point for design comparisons within a specific trial. More time, technical advice and data will be needed to expand the guidance to comprehensively include international emission factors and understand country-specific trial emissions.

It is important to note that the estimated footprint of a trial calculated prospectively may differ from that of the completed trial. Estimating the footprint at the planning stage is intended to enable lower carbon trials by comparison of alternative designs. Footprinting during and at the end of trials is also important, the former as part of trial monitoring if amendments are made, and the latter for sponsors and funders to be able to report on the footprint of their trials portfolio.

### Building a community

The project team (ICR-CTSU and University of Liverpool) have been awarded further NIHR funding to refine and expand the method to source emission factors for more trial activities including laboratory testing (eg, virology and immunology testing), technology use in trials, for example, electronic data collection and storage (ePROs), activity-based emission factors for data linkage and phase I trials.^22^ Work to further assess, refine and improve assumptions such as inclusion of sustenance in the trial-specific meetings and travel module and the footprint of CTU staff emissions, is also planned.

Recognising the growing interest and support for this area of work, the NIHR MRC TMRP convened the ‘Greener Trials’ group in 2023 as a forum to share resources and facilitate consideration and uptake of more responsible research practice in clinical trials. The group awarded funding to the ICR project team to disseminate the method and train the UK and Ireland academic trialist community in carbon footprinting via monthly drop-in clinics, recorded webinars and educational workshops. Trialists interested in attending a drop-in clinic, should email cict-icrctsu@icr.ac.uk. As trials are footprinted and the results shared through this collaboration, the guidance will be updated in line with accumulating data so that it becomes as comprehensive and applicable to as many trials as possible. The more trials that are footprinted the more we will be able to draw conclusions about trial carbon footprints in relation to trial type and design and share best practice. The guidance will also be updated in line with evolving emission factors and future iterations will be published on the TMRP website.

### Next steps

Our study has identified several areas where future work is needed. The project team received interest from several CTUs who decided they did not have the capacity to participate. This illustrates the challenge of making this routine practice in the UK academic clinical trials community. Currently, carbon footprinting takes time and will be difficult to include at the design stage without the appropriate resources and tools, such as an online calculator. The project team is looking to develop a free to use, online eco-design tool tailored to UK academic clinical trials which is aligned and compatible with parallel workstreams underway in the NHS, pharmaceutical industry and internationally (eg, South African Medical Research Council).

The guidance defines the scope of a clinical trial as the emissions associated with activities funded and defined in the protocol. Currently this scope excludes the manufacture of the IMP, device or other intervention. Future collaboration is planned with the Greener NHS and the Getting it Right First Time teams to link and align footprinting initiatives so that the footprint of academic clinical trials can be considered alongside the clinical intervention under investigation.^23^ This work will be critical in understanding the trade-off between the additional footprint of a clinical trial vs the potential carbon increase or saving if the intervention under investigation became the new standard of care.

## supplementary material

10.1136/bmjopen-2024-088600 online supplemental file 1

10.1136/bmjopen-2024-088600 online supplemental file 2

10.1136/bmjopen-2024-088600 online supplemental file 3

## Data Availability

All data relevant to the study are included in the article or uploaded as online supplemental information.
