# Supplementary material for: What is the carbon footprint of academic clinical trials? A study of hotspots in 10 trials
Source: BMJ Open. 2024 Oct 16;14(10):e088600. doi: 10.1136/bmjopen-2024-088600 (PMC11487931; doi:10.1136/bmjopen-2024-088600)
Supplement: online supplemental file 1 [file bmjopen-14-10-s001.pdf]

FUNDED BY NIHR | National Institute for Health and Care Research

LIVERPOOL CLINICAL TRIALS CENTRE LCTC

ICR

Enabling lower carbon clinical trials: A method to quantify the carbon footprint of clinical trials to inform future lower carbon clinical trial design

Activity data questionnaire

This questionnaire has been developed to collect all information required to carbon footprint a clinical trial using the 'NIHR-funded guidance and method to calculate the carbon footprint of a clinical trial'.

The guidance and accompanying 'Data collation quick guide and worksheet', developed by the Institute of Cancer Research and University of Liverpool on behalf of the NIHR-funded Low Carbon Clinical Trials Group, are publicly available for use.

Within the guidance, clinical trial processes are sub-divided into 10 modules and as such there are 10 sections in the questionnaire to complete:

1. Trial set up
2. CTU emissions
3. Trial specific meetings and travel
4. Treatment intervention
5. Data collection and exchange
6. Trial supplies and equipment
7. Trial specific patient assessments
8. Samples
9. Laboratory
10. Trial close out

This list is not exhaustive, and it is expected that further activities and modules may need to be added to account for specialist processes in all clinical trial types. Furthermore, not all sections may be applicable to your trial.

NOTE:

- Please provide your answers or information in an alternative font colour e.g. RED
- Please use best estimates where you are unsure of the exact number
- Only include activities and processes which have been funded and are defined within the trial protocol
- Only include an activity or patient participation where it exceeds or is additional to routine care, where it is required to establish an endpoint, the patient population or eligibility. Use of the trial SOECAT or the costing included in the initial funding application is encouraged to consistently define the investigations considered in addition to routine care and/or part of the research question. For more details in terms of the scope of application, please refer to the 'NIHR-funded guidance and method to calculate the carbon footprint of a clinical trial'
- Future tense has been used throughout as the guidance is intended as a design tool, but this questionnaire can also be used to gather data from completed trials.

[INSERT STUDY NAME]

Please provide a brief description of the trial:

1. Trial Set-up

This section will allow calculation of the emissions attributed to production, provision and postage of documentation to sites or patients. This is the data required for sections 1.1-1.3 of the guidance.

1a. Number of trial participants or recruitment target
1b. Number of sites (NB: It is useful to include a list of sites or potential sites and their postcode to allow calculation of distance to sites)
1c. Postcode of the CTU or 'Trial HQ' (the location of the trial management team coordinating the trial on behalf of the Sponsor)
1d. Considering all trial documentation produced (e.g. Site Investigator File and contents, Site Pharmacy File and contents, CRF Folder and contents, PIS/C and GP letter). What is the total number of pages printed in black and white?
1e. Considering all trial documentation produced (e.g. Site Investigator File and contents, Site Pharmacy File and contents, CRF Folder and contents, PIS/C and GP letter). What is the total number of pages printed in colour?
1f. Considering the Investigator Site Files produced, are physical copies printed at the CTU and sent to the sites by post/courier? If no, skip to 1h.
1g. Considering the Investigator Site Files produced, how many are sent to each site? Please specify if a large Lever Arch folder or a smaller Ring Binder is used.
1h. If an eTMF is used, what is the GB storage for one eTMF folder? How many years will it be stored? (Right click on the folder and select Properties to see the GB. Use a completed trial with a similar number of sites and patients if the information is unavailable).

2. CTU Emissions

This section will allow calculation of the emissions attributed to energy consumption, heating and commuting for the trial team. This is the data required for sections 2.1-2.3 of the guidance.

2a. What is the total staff FTE required for the whole trial duration?<br>E.g. if 3 people work 100%/1 FTE on the trial, another person works 50%/0.5FTE and another works 25%/ 0.25 then this equates to a total of 3.75 FTE on the trial. NB: If there is trial management occurring in countries outside of the UK, please note the total FTE in each country separately.
2b. If staff work from home, please note the % of FTE spent in office and at home e.g. 2 days per week/40% of FTE in office, 60% from home.

2c. If known, for staff commuting please note the mode of transport and the total distance travelled per day (to and from work) for each FTE.
3. Meetings and Travel<br>**This section allows calculation of emissions attributed to trial staff travel, sustenance and hotel stays during the trial. This is the data required for sections 3.1-3.4 of the guidance.**<br>NB: unless more specific data is available, assume staff travel from CTU location to any site visits.
3a. Thinking about **Feasibility visits** for the trial, if conducted are they virtual or in-person?<br>If conducted virtually:<br>i. How many will be conducted<br>ii. On average, how many people will attend<br>iii. What is their duration e.g. 1 hour<br><br>If conducted in person:<br>i. In which sites<br>ii. How many staff will attend from CTU<br>iii. By what mode of transport will staff travel<br>iv. Will an overnight stay be required
3b. Thinking about **Site initiation visits** for the trial, if conducted are they virtual or in-person?<br>If conducted virtually:<br>i. How many will be conducted<br>ii. On average, how many people will attend<br>iii. What is their duration<br><br>If conducted in person:<br>i. In which sites<br>ii. How many staff will attend from CTU<br>iii. By what mode of transport will staff travel<br>iv. Will an overnight stay be required
3c. Thinking about **Monitoring visits** for the trial, if conducted are they virtual or in-person?<br>If conducted virtually:<br>i. How many will be conducted<br>ii. On average, how many people will attend<br>iii. What is their duration e.g. 1 hour<br><br>If conducted in person:<br>i. In which sites and how many visits to each site<br>ii. How many staff will attend from CTU<br>iii. By what mode of transport will staff travel<br>iv. Will an overnight stay be required
**Governance committee meetings for the trial**<br><br>3d. **TMG meetings**<br>If conducted virtually:<br>i. How many will be conducted

ii. On average, how many people will attend
iii. What is their duration

If conducted in person:
i. How many staff will travel and from what general area
ii. By what mode of transport will staff travel
iii. Will an overnight stay be required
iv. Will a lunch be provided on the day, and/or dinner that evening

3e. **TSC meetings**
If conducted virtually:
i. How many will be conducted
ii. On average, how many people will attend
iii. What is their duration

If conducted in person:
i. How many staff will travel and from what general area
ii. By what mode of transport will staff travel
iii. Will an overnight stay be required
iv. Will a lunch be provided on the day, and/or dinner that evening

3f. **DMC meetings**
If conducted virtually:
i. How many will be conducted
ii. On average, how many people will attend
iii. What is their duration

If conducted in person:
i. How many staff will travel and from what general area
ii. By what mode of transport will staff travel
iii. Will an overnight stay be required
iv. Will lunch be provided on the day, and/or dinner that evening

Other
3g. **Audits or inspections**
If conducted virtually:
i. How many will be conducted
ii. On average, how many people will attended
iii. What is their duration

If conducted in person:
i. How many people will travel and from what general area
ii. By what mode of transport will they travel
iii. Will an overnight stay be required
iv. Will a lunch be provided on the day, and/or dinner that evening

3h. **Conferences or scientific meetings**

If attending virtually:<br>i. How many people will attend<br>ii. What is their duration<br><br>If attending in person:<br>i. How many staff will travel and from what general area<br>ii. By what mode of transport will staff travel<br>iii. Will an overnight stay be required<br>iv. Wil lunch be provided on the day, and/or dinner that evening
4. Intervention<br>Please fill out the applicable section(s) for the interventions(s) in the trial<br>**4.1. Physical (IMP)**<br>**4.2. Clinical (non-IMP)**<br>**4.3. Other (not captured above)**<br><br>**This section describes any processes relating to providing and delivering the trial intervention that are over and above routine care and is the data required for sections 4.1.4-4.3.4 of the guidance. Manufacture of intervention is excluded from the scope of analysis.**
**4.1. Physical (IMP)**<br><br>4.1.a. Is the intervention shipped from a manufacturing site to distribution site and/or participating sites, or direct to participant? If no, skip to 4.1.e.
4.1.b. Please provide the locations the intervention is shipped from and to, and the quantity shipped (weight in tonnes if possible).
4.1.c. Is the intervention shipped ambient, refrigerated, or frozen?
4.1.d. What packaging is the intervention shipped in (cardboard, plastic, polystyrene)? Please estimate the weight in kilograms.
4.1.e. Is there a requirement for destruction of overage (e.g. incineration of IMP)? If so, please estimate how much (in kilograms) or a % that will be destroyed.
**4.2. Clinical (Non-IMP)**<br><br>4.2.a. Is there shipping of an intervention, or any materials required to deliver the intervention e.g. from manufacturing site to participating sites, or direct to participant? If no, skip to 4.2.e.
4.2.b. Please provide the locations the intervention is shipped from and to, and the quantity shipped (weight in tonnes if possible).

4.2.c. Was the intervention shipped ambient, refrigerated or frozen?
4.2.d. If feasible to estimate, please provide the weight in kg of each material required for the packaging and shipment of intervention (e.g. cardboard, plastic or polystyrene), or materials required to deliver the intervention.
4.2.e. Please provide detail of any activities or resources required/relating to delivery of the intervention. E.g. if surgery, please provide an estimate of the surgery duration, staff hours required and the number of patients to undergo the surgery.
**4.3. Other (not captured above)**<br><br>4.3.a. Is there shipping of an intervention, or any materials required to deliver the intervention, e.g. from manufacturing site to participating sites, or direct to participant? If no, skip to 4.3.d.
4.3.b Please provide the locations the intervention is shipped from and to, and the quantity shipped (weight in kg or tonnes if possible).
4.3.c. If feasible to estimate, please provide the weight in kg of each material required for the packaging and shipment of intervention (e.g. plastic, cardboard or polystyrene), or materials required to deliver intervention.
4.3.d. Is any travel required to facilitate delivery of the intervention? If so:<br><br>i. Where will people travel from/to (general area)?<br>ii. What mode of transport is likely to be used?<br>iii. Is an overnight stay required?
4.3.e. Please provide detail of any other activities or resources required/relating to delivery of the intervention.
5. Data collection and exchange<br>**This section allows calculation of emissions related to how data is collected and stored. This is the information required for sections 5.1-5.4 of the guidance.**<br><br>NB: analysis of data does not need to be calculated separately, it is covered by the emissions attributed to trial staff FTE in "CTU emissions" and calculations included within "Data Collection and exchange".
5a. Will sites post any paper CRF's back to site? If so, state the number of pages from each site. Please do not include patient questionnaires in this.

5b. Will sites post any CD's back to CTU? If so, state the number.

5c. Can you guesstimate how many emails may be sent during the trial? E.g. 15 emails a day? 50 emails a day?

5d. Do you have an electronic trial database collecting data? If yes, what is the gigabytes of storage for it and how long will it be stored?

5e. Will patients complete any questionnaires or diaries during the study?

If paper:

i. How many pages will one patient post back, on average?
ii. How many patients will post them back?

If electronic:

i. How long do participants require to complete all questionnaires? Please provide your answer in minutes or hours.
ii. How will participants complete the questionnaires e.g. are participants provided with a device or is a study app used?

5f. Do you intend to use data linkage for your study? If so, how much is budgeted?

6. Trial supplies and equipment

This section allows calculation of emissions attributed to equipment used by the trial team, sites and/or patients. This is the data required for sections 6.1-6.3 of the guidance.

6a. How much is spent on computers and/or software for the trial team? This can usually be found in the grant application.

6b. Will any specific equipment be sent to the sites? Please specify. If no, skip to question 6.e.

6c. Where is the equipment shipped from (CTU or manufacturer) and to which sites?

6d. What is the approximate weight of the shipment?

6e. Are participants provided with any equipment? E.g. wearables such as a smart watch or devices such as smartphone or tablet? Please specify.

6f. How many devices will be provided and how long will they be used for?

6g. How will the patients receive the equipment i.e. is it sent direct to participant from CTU or provider, or collected from site?

7. Trial specific patient assessments

This section will allow calculation of emissions attributed to trial assessments. This is the data required for sections 7.1-7.3 of the guidance.

7a. Number of times each participant is required to travel to hospital to attend a study visit (in addition to routine care)? E.g. 3 study visits in a hospital over duration of study.

7b. Number of times each participant is required to travel to a GP to attend a study visit (in addition to standard of care)? e.g. 3 study visits in a GP setting over duration of study.

7c. What is the total hospital staff FTE required for the whole trial duration? This may be listed in the SoECAT or funding application, if the trial pre-dates the use of a SoeCAT provide a best estimate.

7d. What patient assessments are required that are over and above routine care and how many are required per participant? E.g. MRIs, low or high intensity bed days, blood tests, etc.

8. Samples

This section will allow calculation of emissions associated with collecting samples using sample kits. This is the data required for sections 8.1-8.4 of the guidance.

8a. Are sample kits provided? If so, list each component and the quantity.

8b. Are the sample kits put together and shipped from the CTU? If so, where are each of the sample kit materials ordered from?

8c. How many kits will be shipped to each site?

8d. Will sites send samples to the CTU or a central laboratory for storage or analysis? Please provide the location of central lab if so.

8e. How many samples will be shipped from each site to the central laboratory?

8f. Are samples shipped ambient, refrigerated or frozen?

9. Laboratory

This section will allow calculation of emissions attributed to laboratory processes and sample storage at a central laboratory and/or site laboratories. This is the data required for sections 9.1-9.3 of the guidance.

9a. What is the total laboratory staff FTE required for the whole trial duration? The FTE of central lab staff may be found in the funding application, or in the SoECAT for participating site lab staff. Alternatively provide a best estimate.
9b. If there is no central lab analysis, is there any initial processing of samples at site? Please specify number of samples and type of processing (e.g. centrifugation for 20 minutes).
9c. Are samples stored in a fridge or freezer at site and/or central laboratory? Please specify if a ULT freezer.
9d. Considering any storage at site or central laboratory, what is the total time samples will be stored and how much space do they require (e.g. half or a third of a freezer)?
10. Trial close out<br>**This section allows calculation of emissions attributed to storage of samples and documentation after the trial ends. This is the data required for sections 10.1-10.3 of the guidance.**
10a. Roughly how many files or storage boxes are archived?
10b. What type of building will the paper documentation be archived in: office, lab, hospital building or warehouse?
10c. How many years are the files archived for? This is usually stated in the protocol or the IRAS application.
10d. Is there electronic storage of documentation? If so, please provided the GB stored and length of storage (years).
10e. Is there any ambient storage of samples (e.g. microscope slides)? If so:<br>i. What type of building will they be archived in: office, lab, hospital building or warehouse?<br>ii. How much space do you estimate they will require (m^2)?<br>iii. How long will they be archived for in years? This is usually stated in the protocol or the IRAS application.
10f. Are any samples destroyed? If so, how many?
10g. Is there return of any equipment from sites to CTU? If so, provide detail about what is returned and from where.
