# Supplementary material for: What is the carbon footprint of academic clinical trials? A study of hotspots in 10 trials
Source: BMJ Open. 2024 Oct 16;14(10):e088600. doi: 10.1136/bmjopen-2024-088600 (PMC11487931; doi:10.1136/bmjopen-2024-088600)
Supplement: online supplemental file 2 [file bmjopen-14-10-s002.pdf]

FUNDED BY NIHR | National Institute for Health and Care Research

LIVERPOOL CLINICAL TRIALS CENTRE LCTC

ICR

Enabling lower carbon clinical trials: A method to quantify the carbon footprint of clinical trials to inform future lower carbon trial design

Detailed Guidance and method to calculate the carbon footprint of a clinical trial

Background

Almost 17 years ago, the Sustainable Trials Study Group concluded that clinical trials contribute substantially to greenhouse gas emissions, notably through energy use in research premises and air travel[1].

In addition, a study conducted in 2009 of 12 UK pragmatic randomised trials involving an average of 402 participants showed that the average carbon emission generated by the trials was 78.4 tonnes of carbon dioxide equivalent [2]. Multiplying this total by the 350,000 national and international trials registered on ClinicalTrials.gov, this would estimate that emissions attributable to all global clinical trials to be about 27·5 million tonnes of carbon dioxide equivalent[3].

Since then, the urgency of the threat from the climate crisis has increased exponentially and the World Health Organization calls climate change the single biggest health threat facing humanity[4]. Planned climate action is not sufficient to prevent the current warming predictions; humanity must reach net-zero by 2050 to limit warming to 1.5 degrees and avoid the worst consequences of climate change.

As a first step to reduce the environmental impact of clinical trials, a method to quantify the carbon footprint of a clinical trial and associated processes is required.

Introduction

This guidance provides information on how to carbon footprint a clinical trial for the purposes of the NIHR-funded project 'enabling lower carbon clinical trials.'

Within the guidance, clinical trial processes have been sub-divided into the following modules:

1. Trial set up
2. CTU emissions
3. Trial specific meetings and travel
4. Treatment intervention
5. Data collection and exchange
6. Trial Supplies and equipment
7. Trials specific patient assessments
8. Samples
9. Laboratory
10. Trial close out

The above list is not exhaustive, and it is expected that further activities and modules may need to be added to account for specialist processes in all clinical trial types and as knowledge around life cycle analysis increases.

NB: analysis of data does not need to be calculated separately, it is covered by the emissions attributed to trial staff FTE in "CTU emissions" and calculations included within "Data Collection and exchange".

Calculating carbon footprint

A carbon footprint is a measure of greenhouse gases, usually quoted in kg or tonnes of carbon dioxide equivalent (CO_2e). To calculate the carbon footprint of a particular clinical trial process, both 'activity data' and 'emission factors' are required.

An 'activity' could be anything from electricity consumption to materials, travel and food. Activity data quantifies the amount of that activity e.g. distance travelled, kWh used etc.

An emission factor, also known as a conversion factor, "is a coefficient which allows you to convert the activity data into greenhouse gas emissions. It is the average emission rate of a given source, relative to units of activity or process/processes."[5]

To calculate a carbon footprint of a trial, the activity data will need to be provided by the trial management team and multiplied by the emission factors provided in this guidance document. Two types of activity data may be used:

- Primary data: data collected first-hand from specific activities within the studied clinical trial process i.e., the data collected where you can determine the amount of the activity taking place, for example electricity in kWh used by a building or the weight of an IMP shipment and the distance it travels.

- Secondary data: activity data that is not collected from specific activities within the studied clinical trial because you cannot determine or measure the exact quantity of the activity taking place, for example the number of m^2 occupied by an office worker or hospital worker. Secondary data may take the form of average, or typical, information about an activity from a published study or other source and will be provided in this guidance document e.g., average m^2 occupied by an office worker or average distance travelled.

Primary activity data are preferred for all activity data used in each module. However, secondary data may be used where primary activity data is unavailable or difficult to obtain.

It is important to avoid double-counting activities i.e., modules must not include activities already covered by other modules in the clinical trial process map. A data collection tool is provided alongside this guidance to aid in this process and help avoid double-counting.

Scope

This guidance describes a method to calculate the carbon footprint of a UK, academic clinical trial.

It can be applied to trials with international participation, however as emission factors vary between countries, and those provided within this guidance are UK-specific, country-specific emission factors may be required. Proxy emission factors can be used where appropriate and the source country of an emission factor will be stated where applicable.

The tool is intended to calculate the carbon footprint of the activities specific to the clinical trial, defined as the data required to analyse the trial endpoints and the research activities over and above standard of care.

The guidance is intended for use as a tool to inform sustainable decision-making in the design of clinical trials, rather than a tool to calculate the absolute footprint of a clinical trial or compare environmental performance of one trial over another.

Limitations

There are a number of emission factors that can be used for a particular process and activity data can be calculated in a number of ways. Therefore, life cycle analysis produces variation in its results, dependent on choices made by the individual performing the calculations.

We have endeavoured to include an explanation or justification for the choice of emission factors used. In addition, the emission factors have been selected as the most applicable and up to date factors that are freely available for public use. It is important to note that more up to date factors, or forecasted emission factors, may be available, but they are subject to licensing requirements and are not publicly available. The source of all factors used is included for reference.

This guidance accounts only for the greenhouse gas emissions. It does not include other metrics that are also important to consider when evaluating sustainability and the potential trade-offs, for example water use, land use, waste and those relating to social and economic impacts.

Assumptions

- The eventual aim of this tool is to be used prospectively during the design phase of a trial, before trial funding is secured. However, the tool will not capture the carbon footprint associated with work conducted during this period i.e., prior to confirmation of funding award. The tool can also be used retrospectively on clinical trials which are complete.
- The tool only calculates emissions of processes which have been funded and defined within the trial protocol i.e., future planned work which has not yet been funded or that will be defined outside of the protocol are not included.
- The tool only calculates the emissions of patient participation where it exceeds or is additional to routine care, where it is required to establish an endpoint, the patient population or eligibility. Use of the trial SOECAT or the costing included in the initial funding application (if the trial predates use of SOECAT) is encouraged, to consistently define the investigations considered in addition to routine care and/or part of the research question.
- For all translational/optional/research samples and sub studies, the tool does not calculate emissions associated with analysis performed by central laboratories or collaborators, but does calculate emissions for activities defined in the protocol, such as collecting the sample or data from patients, initial processing at participating sites and shipment of samples or data to the site of subsequent analysis.
- This guidance will only appraise trials with UK based trial management (sites may be international but the trial must be overseen by a UK Clinical Trials Unit/Sponsor/ Research team).
- The carbon footprint associated with the manufacture of a trial intervention e.g. Investigational Medicinal Products (IMPs) and medical devices is not included in this guidance.
- The tool does not calculate the carbon footprint of waste associated with a particular clinical trial. Concerning clinical trial consumables, activity data is based on the quantities purchased. The only exception to this is the destruction of unused IMP at participating sites, as this is an activity specifically undertaken for a clinical trial which has an associated carbon footprint.
- The tool does not calculate the carbon footprint of hospital and laboratory staff commuting.
- Carbon emissions generated by ethics and regulatory approval bodies are not within the scope of this guidance and will not be calculated.

NB: more module specific assumptions can be found throughout the document where applicable.

1. Trial set up

This module includes the following activities:

1.1 Production of trial documentation to be sent to sites or participants
1.2 Provision/postage of trial documentation to sites
1.3 Provision/postage of documentation to participants by CTU or participating sites
1.4 Provision/postage of incentives to participant

1.1. Production of trial documentation

For production of trial documentation, the carbon footprint of both the printing and materials must be calculated.

Printing: The number of pages must be multiplied by 0.005 to produce a weight in kilograms which is then multiplied by the relevant emission factor provided below.

- Black and white: kg (paper) x 0.22438 = kgCO_2e
- Colour: kg (paper) x 0.31786 = kgCO_2e

1292	use, printer, laser jet, b/w, per kg printed paper	electronics	services	kg	RER	0.22438
1293	use, printer, laser jet, b/w, printing per h	electronics	services	h	CH	0.37213
1294	use, printer, laser jet, b/w, printing per h	electronics	services	h	RER	0.39065
1295	use, printer, laser jet, colour, per kg printed paper	electronics	services	kg	CH	0.2027
1296	use, printer, laser jet, colour, per kg printed paper	electronics	services	kg	RER	0.31786

NB**:** If you are unable to calculate the number of pages, you may assume that there are 150 pages in a small ring binder and 500 pages in a large lever arch folder.

Assumption: 1 piece of paper weighs around 5g/0.005kg

Emission factor source: Ecoinvent, version 2.2, 2011 [6] (RER = European emission factor)

Materials (paper): For the carbon footprint of paper production**,** the number of pages must be multiplied by 0.005 to produce a weight, and the weight multiplied by the emission factor for paper manufacture provided below.

Paper emission factor: 0.91048 kg CO_2e per kg of paper

Calculation: kg (paper) x 0.91048 = kg CO_2e

Emission factor source: Greenhouse gas reporting: conversion factors 2023, GOV.UK [7]

Materials (folders): As trial documentation is often sent to sites in folders, provided below are estimated weights of folders. The total weight in kg will then need be multiplied by the emission factor for cardboard (provided below).

Board emission factor: 0.8015 kg CO_2e per kg of cardboard

Calculation: Kg (cardboard) x 0.8015 = kg CO_2e

Lever arch: Assumption: Weight of lever arch = 0.5kg

Ring binder: Assumption: Weight of ring binder = 0.3kg [Eastlight A4 Black Ring Binder - EA54121 (staples.co.uk)]

Emission factor source: Greenhouse gas reporting: conversion factors 2023, GOV.UK [7]

1.2, 1.3, 1.4 Provision of trial materials

For provision of trial materials to sites by post/courier you will need to calculate the total weight of the materials (in tonnes) and multiply this by the distance they travel (in kilometres) to get tonne.km. The t.km is then multiplied by the emission factor for either road or air freight provided below.

Road freight:

Mass of freight (tonnes) x distance (km) = t.km

t.km x emission factor = (kg CO_2e)

Emission factor for road freight = 0.19443

1942	2724 transport, lorry 20-28t, fleet average	transport systems	road	tkm	CH	0.19443

Assumption: If unknown, for delivery of trial supplies to patients or GP, use 17.4km as distance from hospital to patient, or hospital to GP in the UK. Source: BMJ 2009;339:b4187 [2]

Emission factor source: Ecoinvent, version 2.2, 2011 [6]

Air freight:

Mass of freight (tonnes) x distance (km) = t.km

t.km x most suitable emission factor from the below table = (kg CO_2e)

Activity	Type	Unit	kg CO_2e
Freight flights	Domestic, to/from UK	tonne.km	4.673396
	Short-haul, to/from UK	tonne.km	1.668155
	Long-haul, to/from UK	tonne.km	1.099032
	International, to/from non-UK	tonne.km	1.099032

For air freight you must also add the well-to-tank (WTT) value to get the final total. WTT refers to the emissions attributed to production, transportation and distribution of vehicle fuel.

WTT can be calculated by multiplying the t.km used in the first calculation by the correlating WTT conversion factor provided below.

Activity	Type	Unit	kg CO_2e
WTT- freight flights	Domestic, to/from UK	tonne.km	0.57429
	Short-haul, to/from UK	tonne.km	0.20515
	Long-haul, to/from UK	tonne.km	0.13516
	International, to/from non-UK	tonne.km	0.13516

Calculation: t.km x correlating WTT conversion factor

NB: 'Short-haul' is considered as international flights to/from the UK that are up to 3700km distance. 'Long-haul' is considered as international flights to/from the UK that are over 3700km distance. The 'International' emission factor can be used where flights are between non-UK countries.

NB: The 'With RF' values have been provided. RF (combustion and radiative forcing) includes the indirect and direct emissions.

Emission factor source: Greenhouse gas reporting: conversion factors 2023, GOV.UK [7]

2. CTU Emissions

This module includes the following activities:

2.1. Energy consumption at CTU
2.2. Heating
2.3. Trial team commuting

2.1. Energy consumption at CTU (according to staff FTE)

It can be difficult to estimate the carbon footprint associated with energy consumption by a CTU because the space or building may be used for other trials not being appraised and non-trial activities. The method described below therefore estimates emissions per employee based on average statistics and benchmarks.

According to the UK Employment Destiny Guide, public sector office space is 12 m^2 per FTE. This is multiplied by 68 kWh (the median electricity intensity for offices per m^2) to produce a per person per year usage. The emissions attributed to CTU energy consumption can then be calculated by multiplying the per person usage by the electricity emission factor. The UK electricity emission factor and the calculation are provided below.

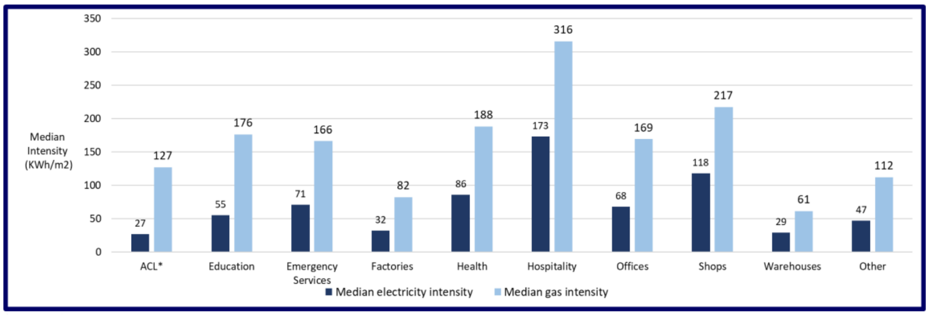

2023 UK electricity emission factor = 0.257 kg CO_2e per kWh

Calculation

12m^2 x 68 kWh/m^2 = 816 kWh per FTE per year

816 kWh x 0.257 = 209.7 kgCO_2e per FTE per year

Multiply 209.7 kgCO_2e by the FTE required for the whole trial duration.

Assumption: According to the UK EMPLOYMENT DENSITY GUIDE, 3rd edition November 2015, office space per FTE is 12 m^2 (public sector) [8]

Office benchmark data source: The Non-Domestic National Energy Efficiency Data-Framework 2023 (England and Wales) (publishing.service.gov.uk) [9]

Electricity emission factor source: Greenhouse gas reporting: conversion factors 2023, GOV.UK [7]

2.2 Heating and Homeworking

For heating, the calculation follows the same method as above. The 12m^2 per person is multiplied by the office heating benchmark and then by the natural gas conversion factor provided below.

Office building heating benchmark: **169** (kWh/m^2)

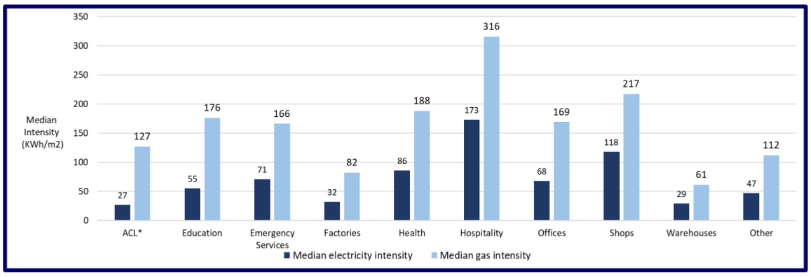

2023 UK Natural gas conversion factor: **0.213**

Calculation

- 12 m^2 x 169 kWh/m^2 = 2028 kWh per FTE per year
- 2028 kWh x 0.213 = 432 kg CO_2e per FTE per year

Multiply 432 kg CO_2e by the FTE required for the whole trial duration.

Assumption: If the heating source is unknown, assume natural gas.

Assumption: According to the UK EMPLOYMENT DENSITY GUIDE, 3rd edition November 2015, office space per FTE is 12 m^2 public sector. [8]

Office benchmark data source: The Non-Domestic National Energy Efficiency Data-Framework 2023 (England and Wales) (publishing.service.gov.uk) [9]

Natural gas emission factor source: Greenhouse gas reporting: conversion factors 2023, GOV.UK [7]

Homeworking

Homeworking (office equipment + heating)	per FTE Working Hour	0.33378

Multiply the total number of working hours by the conversion factor (0.33378) to calculate kgCO_2e (includes electricity use from office equipment and heating).

NB: You may assume 1 FTE is equal to 1800 hours. This was calculated based on an 8-hour working day and 225 working days per year (260 working days in a year minus 35 days paid leave and sickness).

Emission factor source: Greenhouse gas reporting: conversion factors 2023, GOV.UK [7]

2.3. Trial team commuting

The emissions attributed to the trial team commuting can be calculated either using primary data if commuting distance and mode of transport are known, or using secondary data, i.e. average commuting statistics, if primary data is unavailable.

Commuting calculation using primary data:

For cars and motorbikes, multiply the distance travelled (kilometres) by a vehicle by the relevant emission factor below:

- Average petrol car: 0.209419 kg CO_2e per km
- Average diesel car: 0.211276 kg CO_2e per km
- Average hybrid car: 0.150069 kg CO_2e per km
- Average motorbike: 0.143234 kg CO_2e per km

NB: The above are vehicle.km emission factors (emissions are attributed to the whole vehicle).
NB: WTT has been included in the values.

For public transport such as buses and trains, multiply the distance travelled (kilometres) by a passenger by the relevant emission factor below:

- National rail: 0.044433 kg CO_2e per p.km
- London Underground: 0.035082 kg CO_2e per p.km
- Light rail/tram: 0.036093 kg CO_2e per p.km
- Local bus (not London): 0.147233 kg CO_2e per p.km
- London Bus: 0.097483 kg CO_2e per p.km

NB: The above are passenger.km emission factors (emissions are attributed on a single-person basis).
NB: WTT has been included in the values.

Emission factor source: GOV.UK Greenhouse gas reporting, conversion factors 2023 [7]

2.3. Trial team commuting (continued)

Calculation using average commuting statistics

The average distance of a commuting journey was 8.8 miles (14km) in 2013/14. Multiplied by 225 commuting days, this results in a total distance of 6300 km travelled per year. UK government travel statistics have been used to identify the percentage each mode of transport is used to commute. This percentage has been multiplied by the relevant emission factor below, and all modes of transport have been added to produce an average kg CO_2e per FTE per year.

- **Car**
  - 68% of people commute by car
  - 68% of 6300 km = 4284 km
  - 4284km x 0.209419 = 897.15 kgCO_2e
  - NB: average petrol car emission factor used
- **Rail**
  - 9% of people commute by rail
  - 9% of 6300 km = 567 km
  - 567 km x 0.038536 = 21.85 kgCO_2e
  - NB: emission factor includes national rail, light rail/tram and London underground
- **Bus**
  - 6% of people commute by bus
  - 6% of 6300 km = 378 km
  - 378 km x 0.127 = 48 kgCO_2e
- **Walk**
  - 11% of people walk their commute, therefore 693km = 0 kgCO_2e
- **Other**
  - 5% of people commute by other means (bicycle, motorcycle and taxi)
  - Assume 3% cycling therefore zero emissions
  - 1% motorbike: 63 km x 0.143234 = 9 kgCO_2e
  - 1% taxi: 63km x 0.185585 = 11.7 kgCO_2e

Total emissions attributed to **1 FTE commuting for 1 year = 987.7 kgCO_2e**

Multiply by the number of years and FTE applicable

NB: The WTT (well-to-tank) has been included in the emission factors for all modes of transport and therefore does not need to be added.

Assumptions:

- 1 FTE = 225 days spent commuting. This was calculated by subtracting 7 sick days and 28 days paid leave from the 260 workdays in a year.
- 6300 km is the total distance an employee will commute per year. This was calculated by multiplying 28km (14km per commuting journey) by 225 days.

Benchmark data and emission factor sources:

- Transport Statistics Great Britain: 2022 Domestic Travel - GOV.UK (www.gov.uk) [10]
- Commuting trends in England 1988 - 2015 (publishing.service.gov.uk) [11]
- Emission factor source: GOV.UK Greenhouse gas reporting, conversion factors 2023 [7]

3. Trial specific meetings and travel

This module includes the following activities:

- **3.1. Visits and travel to site**
- **3.2. Travel to meetings**
- **3.3. Hotel stays**
- **3.4. Sustenance**

3.1, 3.2. Visits and travel to site, travel to meetings

Rail, bus and taxi

For business travel by bus, taxi and rail, the activity data is captured in passenger km (p.km). The number of passengers is multiplied by the distance travelled (km), then by the relevant emission factor provided below.

Emission factors:

- National rail: 0.044433 kg CO_2e per p.km
- London Underground: 0.035082 kg CO_2e per p.km
- Light rail/tram: 0.036093 kg CO_2e per p.km
- International rail: 0.005629 kg CO_2e per p.km
- Local bus (not London): 0.147233 kg CO_2e per p.km
- London Bus: 0.097483 kg CO_2e per p.km
- Regular taxi: 0.185585 per p.km

Calculation:

Number of passengers x total distance (km) = p.km

p.km x emission factor = (kg CO_2e)

NB: All emission factors provided relate to ‘kgCO_2e’ and include WTT.
NB: Distances can be calculated using google maps, remember to include the return journey.
NB: Unless more specific data is available, assume staff travelled from CTU location to any site visits.

Emission factor source: Greenhouse gas reporting: conversion factors 2023, GOV.UK [7]

Car

For business travel by car, the activity data is captured in vehicle km (v.km). The number of vehicles is multiplied by the distance travelled (km), then by the relevant emission factor.

- Average petrol car: 0.209419 kg CO_2e per km
- Average diesel car: 0.211276 kg CO_2e per km
- Average hybrid car: 0.150069 kg CO_2e per km

NB: All emission factors provided relate to ‘kgCO_2e’ and include WTT.
NB: Distances can be calculated using google maps, remember to include the return journey.
NB: Unless more specific data is available, assume staff travelled from CTU location to any site visits.
NB: Assume average petrol car if unknown.

Emission factor source: Greenhouse gas reporting: conversion factors 2023, GOV.UK [7]

3.1, 3.2. Visits and travel to site, travel to meetings: Air travel

For business travel by air, activity data is also captured in passenger km (p.km). The number of passengers is multiplied by the distance travelled (km), then by the relevant emission factor.

Number of passengers x total distance (km) = p.km

p.km x suitable emission factor = (kg CO_2e)

An emission factor must be chosen from the following categories:

- Domestic
- Short Haul International (≤3700 km) – Average/Economy/Business
- Long Haul International (>3700 km) – Average/Economy/Business
- International (travel between non-UK countries)

Activity	Haul	Class	Unit	kg CO_2e
Flights	Domestic, to/from UK	Average passenger	passenger.km	0.27258
	Short-haul, to/from UK	Average passenger	passenger.km	0.18592
		Economy class	passenger.km	0.18287
		Business class	passenger.km	0.27430
	Long-haul, to/from UK	Average passenger	passenger.km	0.26128
		Economy class	passenger.km	0.20011
		Premium economy class	passenger.km	0.32016
		Business class	passenger.km	0.58029
		First class	passenger.km	0.80040
	International, to/from non-UK	Average passenger	passenger.km	0.17580
		Economy class	passenger.km	0.13464
		Premium economy class	passenger.km	0.21542
		Business class	passenger.km	0.39044
		First class	passenger.km	0.53854

For business travel you need to add the WTT (well-to-tank) value to get the final total. WTT can be calculated by multiplying the p.km used in the first calculation by the correlating WTT conversion factor provided below.

				With RF
Activity	Haul	Class	Unit	kg CO_2e
WTT- flights	Domestic, to/from UK	Average passenger	passenger.km	0.03350
	Short-haul, to/from UK	Average passenger	passenger.km	0.02286
		Economy class	passenger.km	0.02249
		Business class	passenger.km	0.03373
	Long-haul, to/from UK	Average passenger	passenger.km	0.03213
		Economy class	passenger.km	0.02461
		Premium economy class	passenger.km	0.03937
		Business class	passenger.km	0.07137
		First class	passenger.km	0.09844
	International, to/from non-UK	Average passenger	passenger.km	0.02162
		Economy class	passenger.km	0.01656
		Premium economy class	passenger.km	0.02649
		Business class	passenger.km	0.04802
		First class	passenger.km	0.06623

NB: Values relating to 'kgCO_2e' provided.
NB: Distances can be calculated using google maps.
NB: 'With RF' values are provided. RF (combustion and radiative forcing) includes the indirect and direct emissions.

Assumption: travellers departed from the nearest airport to their place of work and flew directly to the airport of the city to which they were travelling.

Emission factor source: Greenhouse gas reporting: conversion factors 2023, GOV.UK [7]

3.2 Travel to meetings: Teleconferencing

For meetings which are conducted by teleconferencing, multiply the number of people and hours by the figure provided below.

Videoconferencing with camera on = 0.1573 kg CO_2e per person per hour.

Videoconferencing with camera switched off = 0.0063 kg CO_2e per person per hour.

Emission factor source: Turn off that camera during virtual meetings, environmental study says - Purdue University News [12]

3.3 Hotel Stays

To calculate the emissions attributed to hotel stays, the number of hotel rooms is multiplied by the length of stay (in number of nights) and by the conversion factor for the appropriate country.

Each country has a different emission factor. Emission factors for the UK are shown below, other countries can be found at ghg-conversion-factors-2023-full-file-update.xlsx (live.com).

Activity	Country	Unit	kg CO_2e
Hotel stay	UK	Room per night	10.4
	UK (London)	Room per night	11.5

NB: A 'room per night' accounts for use of the room and does not differentiate for number of travellers staying in the room.

Emission factor source: Greenhouse gas reporting: conversion factors 2023, GOV.UK [7]

Ideally the above method is used to calculate emissions attributed to hotel stays. However, if the relevant information is unavailable, you may use a cost-based method by multiplying the total cost allocated to hotel stays in the funding application by the emission factor below.

Cost (£) x 0.358 = kg CO_2e

Wholesale distribution	Gov.UK	2020	converted fron £	0.375
Retail distribution	Gov.UK	2020	converted fron £	0.277
Hotels, catering, pubs e	Gov.UK	2020	converted fron £	0.358
Railway transport[5]	Gov.UK	2020	converted fron £	0.678
Road transport[5]	Gov.UK	2020	converted fron £	0.690
Water transport[5]	Gov.UK	2020	converted fron £	1.428
Air transport[5]	Gov.UK	2020	converted fron £	2.089

Emission factor source: Gov.UK Government conversion factors for company reporting of greenhouse gas emissions 2012 - Annex 13 with consideration of 2020 inflation rates. [13]

3.4. Sustenance

For the carbon footprint associated with meeting lunches or hotel dinners, multiply the quantity by the relevant emission factor provided below.

Meeting lunches or hotel dinners (vegetarian) = 2.6 kg CO_2e per meal per person

Meeting lunches or hotel dinners (meat) = 5.92 kg CO_2e per meal per person

Emission factor source = WWF, 2018 Food_in_a_warming_world_report.PDF [14]

4. Intervention

This module includes guidance on the following different types of intervention. Pick the most applicable intervention type from:

4.1 Physical (IMP)
4.2 Clinical (Non-IMP)
4.3 Other (not captured above)

4.1 Physical (an IMP)

4.1.1 Movement of IMP from manufacturing site to distribution/packaging site
4.1.2 Movement of IMP from distribution/packaging site to participating sites or direct to participant
4.1.3 Materials required for the packaging and shipment of IMP
4.1.4 Activities or resources required/relating to delivery of the intervention
4.1.5 Destruction of overage

Assumptions: Calculations do not include manufacture of IMP.

4.2 Clinical (non-IMP)

NB: not all calculations will be relevant to all interventions.

4.2.1 Movement (shipment) of the intervention, or resources required to deliver the intervention
4.2.2 Materials required for the shipment of the intervention
4.2.3 Utilities required for delivery of the intervention
4.2.4 Activities or resources required/relating to delivery of the intervention

Assumptions: Calculations do not include manufacture of device/machinery/equipment delivering the intervention.

4.3 Other (Not captured above)

NB: not all calculations will be relevant to all interventions.

4.3.1 Movement of the intervention to the participant or participating site
4.3.2 Materials required for packaging and shipment of the intervention
4.3.3 Materials or resources required for delivery of the intervention
4.3.4 Travel required to facilitate delivery of the intervention

4.1. Physical (an IMP)

4.1.1.,4.1.2. Movement of intervention to participating site or direct to participant

For road and air freight, please refer to section 1.2, 1.3, 1.4 for the calculation and emission factors. If the delivery is not ambient, follow the guidance provided below.

Refrigerated freight

Increase the total kg CO_2e associated with freight by 15% for samples transported at temperatures of 2-8 degrees.

Frozen (dry ice) freight

When calculating the carbon footprint of frozen shipments, make sure to consider the emissions attributable to the dry ice, both in terms of:

1. Weight: If the total weight of the posted package is not available, when estimating the weight of the sample and box, make sure to include the additional weight due to the dry ice (add/include in normal calculation of weight x distance x emission factor).
2. Emissions of dry ice manufacture: For 1 kg of dry ice, you need to account for 2.22 kg of liquid CO_2 using the Ecoinvent 2.2 data below. 2.22kg x 0.81605 = **1.81 kg CO_2e per 1 kg dry ice** produced/used.

261	443	carbon black, at plant	chemicals	inorganics	kg	GLO	2.3658
262	444	carbon dioxide liquid, at plant	chemicals	inorganics	kg	RER	0.81605
263	445	carbon monoxide, CO, at plant	chemicals	inorganics	kg	RER	1.5539
6949	446	cerium concentrate, 60% cerium oxide, at plant	chemicals	inorganics	kg	CN	8.309

NB: If the amount of dry ice used in frozen shipments is unknown, estimate 1kg of dry ice per sample box. Ensure this additional weight is included in the freight calculation.

Emission factor source: Consultation /estimation by Environmental Resource Management

4.1.1., 4.1.2 Movement of intervention to participating site or direct to participant (continued)

Sea freight

For transport of an intervention via cargo ship, you will need to calculate the total weight of the freight (in tonnes) and multiply this by the distance travelled (in kilometres) to get tonne.km. The t.km is then multiplied by the most relevant emission factor provided below:

- Emission factor for freight via 'average container ship' = 0.01977 kg CO_2e per t.km
- Emission factor for freight via 'average RoRo-Ferry' = 0.06328 kg CO_2e per t.km

NB: a RoRo-Ferry is a ship which allows easy loading and disembarking of vehicles carrying freight.
NB: All emission factors provided relate to 'kg CO_2e' and include WTT.

Calculation:

Mass of freight (tonnes) x distance (km) = t.km

t.km x emission factor = (kg CO_2e)

Sea freight emission factor source: Greenhouse gas reporting: conversion factors 2023, GOV.UK [7]

4.1.3. Materials involved in the packaging and shipment of the intervention

Shipping boxes

For single use (SU) cold storage boxes multiply the number required by 25.2 kg CO_2e

For reusable cold storage boxes, multiply the number required by 2.2 kg CO_2e

Sample Average Shipping Box (85% ambient / 15% frozen)	1.34	kg CO_2e	per cold storage box	The International Journal of Life Cycle Assessment, Goellner et al. Vol 19, pp 611–619 (2014)
Sample Cold Storage Box Manufacture (SU)	25.2	kg CO_2e	per cold storage box	The International Journal of Life Cycle Assessment, Goellner et al. Vol 19, pp 611–619 (2014)
Sample Cold Storage Box Manufacture (Reuse)	2.2	kg CO_2e	per cold storage box	The International Journal of Life Cycle Assessment, Goellner et al. Vol 19, pp 611–619 (2014)

Emission factor source: The International Journal of Life Cycle Assessment, Goellner et al. Vol 19, pp 611-619 (2014) [15]

For cardboard: Kg (cardboard) x 0.8015 = kg CO_2e

For polystyrene: kg (polystyrene) x 3.76 = kg CO_2e

Emission factor source: Greenhouse gas reporting: conversion factors 2023, GOV.UK [7]

4.1.4. Activities or resources required/relating to delivery of the intervention

For IMP preparation or release, please refer to section 7.3 to calculate the emissions attributed to hospital or pharmacy staff time.

Please refer to section 7.2 for activities that may be relevant to the delivery of the intervention, e.g. a low intensity bed day, but please take care to avoid double counting.

For the carbon footprint of materials (e.g. plastic, paper, glass), please refer to section 8.1.

4.1.5. Destruction of overage

For the destruction of overage, such as the incineration of IMP, multiply the weight in kg of the material being destroyed by the emission factor provided below.

Kg of waste x 2.4252 = kgCO_2e

2958	disposal, hazardous waste, 25% water, to hazardous waste incineration	waste management	hazardous waste incineration	kg	CH	2.4252

Emission factor source: Ecoinvent, version 2.2, 2011 (CH = SWITZERLAND) [6]

4.2. Clinical (e.g. radiotherapy, device, surgery)

NB: not all calculations will be relevant to all interventions. **This section of the method will be further developed as we carbon footprint more trials, so please inform us via CICT-icrctsu@icr.ac.uk if your protocol specifies an activity that has not been included, and we will help to determine the associated carbon footprint.**

4.2.1 Movement of intervention, or materials required to deliver the intervention

Please refer to section 1.2 and 4.1.1, 4.1.2.

4.2.2 Materials involved in the shipment of the intervention

Please refer to section 4.1.3 or 8.1.

4.2.3 Utilities required for delivery of the intervention

Please refer to section 7.3 to calculate the emissions attributed to hospital utilities if required to deliver the intervention.

4.2.4 Activities or resources required/relating to delivery of the intervention

Please refer to section 7.2 for consumables, surgery and other activities that may be relevant to the delivery of the intervention, but please take care to avoid double counting.

To calculate the emissions attributed to incineration, please refer to section 4.1.4.

4.3. Other

NB: not all calculations will be relevant to all interventions. **This section of the method will be further developed as we carbon footprint more trials, so please inform us via CICT-icrctsu@icr.ac.uk if your protocol specifies an activity that has not been included, and we will help to determine the associated carbon footprint.**

4.3.1 Movement of intervention, or materials required to deliver the intervention

Please refer to section 1.2 and 4.1.1, 4.1.2.

4.3.2 Materials required for packaging and shipment of the intervention

Please refer to section 4.1.3 or 8.1.

4.3.3 Materials or resources required for delivery of the intervention

For printing and paper, please refer to section 1.1.

4.3.4 Travel required to facilitate delivery of the intervention

Please refer to section 3.1, 3.2 for travel.

5 Data collection and exchange

This module includes the following activities:

- **5.1. Data collection and query exchange between CTU and sites**
- **5.2. Data sent direct from participants to CTU or participating sites**
- **5.3. Data from labs to CTU**
- **5.4. Data from other collaborators to CTU**

NB: Analysis of data does not need to be calculated separately, it is covered by the emissions attributed to trial staff FTE in "CTU emissions" and by calculations within "Data Collection and exchange".

5.1. Data collection and query exchange between CTU and sites

CRFs

For postage of paper CRFs, please refer to section 1.2 (freight).

Web-based data entry at sites, e.g. CRF completion, will be accounted for by the time a hospital worker spends on the trial and the carbon footprint of the trial databases (See 5.2).

Scans copied to CD

To estimate the emissions attributed to copying patient scans to a CD, add the carbon footprint of CD manufacture to the carbon footprint of computer use.

The carbon footprint of manufacturing a CD = 0.83 kg $C0_2e$ per CD

Emission factor source: Journal of Industrial Ecology, "The Energy and Climate Change Impacts Of Different Music Delivery Methods". Weber et al. Vol 14, Issue 5, pg. 754-769 (2010) [16]

The carbon footprint of copying the scans on to a CD using a computer = 0.18079 kg $C0_2e$ per hour

1269	use, computer, desktop with LCD monitor, active mode	electronics	services	h	RER	0.18079

Emission factor source: Ecoinvent, version 2.2, 2011 [6]

Email traffic

An email without an attachment = 10g CO_2e. Double this for an email with a one-megabyte attachment.

NB: this is an estimate of all emails exchanged between CTU and participating sites throughout the study lifetime, including data query resolution emails.

Emission factor source: Carbon footprint of your emails | mail.com blog [17]

5.1. (continued)

Data collection via electronic trial databases/systems

The combination of transmitting data and storing it in a data centre requires between 3 to 7 kWh per gigabyte. Therefore multiply 5 kWh by the electricity emission factor (0.257) to calculate the kg CO_2e per GB per year.

5 kwh x 0.257 = 1.285 kg CO_2e per GB per year

Assumption: data storage requires 5 kWh.

Emission factor source: Costenaro, D. and Duer, A. The Megawatts behind Your Megabytes: Going from Data-Center to Desktop. [18]

5.3. Data sent direct from participants to CTU/participating sites

For paper questionnaires, please refer to section 1.1. for the carbon footprint of producing the materials and section 1.2. for postage (freight).

Electronic questionnaire

For completion of an electronic questionnaire, you must account for both the use of a device to complete the questionnaire and the carbon footprint of data storage and transmission associated with web surfing.

For completion using a **desktop computer**: 0.18079 kg CO_2e per hour

1269 use, computer, desktop with LCD monitor, active mode	electronics	services	h	RER	0.18079

For completion using a **laptop**: 0.028719 kg CO_2e per hour

1284 use, computer, laptop, active mode	electronics	services	h	RER	0.028719

Computer and laptop emission factor source: Ecoinvent, version 2.2, 2011 [6]

For completion using a **tablet**: 0.027397 kg CO_2e per hour

For completion using a **smartphone**: 0.015068 kg CO_2e per hour

Tablet and smartphone emission factor source: Examining the Carbon Footprint of Devices - Sustainable Software (microsoft.com) [19]

Web surfing = 9.441 g CO2e/hr (10 mins = 1.57 g CO_2e)

Emission factor source: Resources, Conservation and Recycling, "The overlooked environmental footprint of increasing Internet use". Obringer et al. Vol 167 (2021) [20]

5.4. Data from labs to CTU

For data collection via electronic trial database systems estimate 1.285 kgCO_2e per GB per year.

Emission factor source: Costenaro, D. and Duer, A. The Megawatts behind Your Megabytes: Going from Data-Center to Desktop. [18]

5.5. Data from other collaborators to CTU

Data linkage

For data linkage, multiple the cost spent by the emission factor provided below.

Computer services: 0.149 x spend (£) = kg CO_2e

42	Real estate activities	Gov.UK	2020	converted fron	£					0.085
43	Renting of machinery e	Gov.UK	2020	converted fron	£					0.232
44	Computer services	Gov.UK	2020	converted fron	£					0.149
45	Research and developn	Gov.UK	2020	converted fron	£					0.216
46	Legal, consultancy and	Gov.UK	2020	converted fron	£					0.121

Emission factor source: Gov.UK Government conversion factors for company reporting of greenhouse gas emissions 2012 - Annex 13 with consideration of 2020 inflation rates. [13]

6 Trial supplies and equipment

This module includes the following activities:

6.1. Equipment used by CTU
6.2. Equipment and supplies used by participating sites supplied by CTU
6.3. Equipment and supplies provided to participants specifically for the trial

6.1. Equipment used by CTU

The average carbon footprint of a laptop = 422.5 Kg CO_2e (this includes the carbon emissions during the production, transportation and first 4 years of use).

Emission factor source: What Is The Carbon Footprint Of A Laptop? - Circular Computing™ [21]

For any other office machinery and computers purchased for the trial, multiply the cost by the emission factor provided below.

£ x 0.387 = $kgCO_2e$

17	Machinery and equipme	Gov.UK	2020 converted from £	0.512
18	Office machinery and co	Gov.UK	2020 converted from £	0.387
19	Electrical machinery	Gov.UK	2020 converted from £	0.452

Emission factor source: Gov.UK Government conversion factors for company reporting of greenhouse gas emissions 2012 - Annex 13 with consideration of 2020 inflation rates. [13]

6.2. Equipment and supplies used by participating sites supplied by CTU

For the shipment of equipment to participating sites, please refer to section 1.2.

Please inform us via cict-icrctsu@icr.ac.uk if your protocol specifies any equipment or supplies that have not been included and we will help to determine the associated carbon footprint.

6.3. Equipment and supplies provided to participants specifically for the trial

Smartphone: For a smartphone, account for 55 $kgCO_2e$ from manufacture and add 5.5 $kgCO_2e$ per year of usage.

Emission factor source: Examining the Carbon Footprint of Devices - Sustainable Software (microsoft.com) [19]

Tablet: For a tablet, account for 119 $kgCO_2e$ from manufacture and add 10kg CO_2e per year of usage. Assume a maximum lifetime of 3 years, therefore 30 kg CO_2e is the total possible carbon footprint that can be attributed to use.

Emission factor source: Examining the Carbon Footprint of Devices - Sustainable Software (microsoft.com) [19]

Wearables/smart watch: For a smart watch, account for 30.1 kg CO_2e for manufacture and add 1.633 kg CO_2e per year of usage. Assume a maximum lifetime of 3 years, therefore 4.9 kg CO_2e is the total possible carbon footprint that can be attributed to use.

Emission factor source: Apple Watch SE Product Environmental Report [22]

To calculate the carbon footprint associated with shipment of the devices, please refer to section 1.2.

6.3. Equipment and supplies provided to participants specifically for the trial (continued)

The carbon footprint of an **upper arm automatic blood pressure monitor** (manufacture) = 28.2 kgCO_2e.

Considering a 3-year product lifetime, make sure to attribute emissions based on usage specifically for the trial, i.e. if a monitor is only used in a trial for 1.5 years, attribute 14.1 kgCO_2e per device to the trial.

Emission factor source: The Carbon Catalogue public database – Carbon footprints of 866 commercial products across 8 industry sectors and 5 continents (figshare.com) [23]

To calculate the carbon footprint associated with shipment of the devices, please refer to section 1.2.

7 Trial specific patient assessments

This module includes the following activities:

7.1. Travel of patients for study in visits in addition to standard of care (eligibility/screening assessments, trial-specific assessments and procedures)
7.2. Materials and activities required for study assessments in addition to standard of care
7.3. Utilities required for study assessments according to trial staff FTE

7.1. Travel of patients for study in visits that are in addition to standard of care (eligibility/screening assessments, trial-specific assessments and procedures)

If primary data (mode of transport and distance travelled) is available, please refer to section 3.1 for instructions on how to calculate emissions attributed to patient travel. If not, please use the secondary data available below.

Patient travel to elective care (e.g. Hospital)

Emissions associated with 1 visit to elective care (2 journeys – out and back) = 5.8 kgCO_2e

Patient travel to primary care (e.g. GP)

Emissions associated with 1 visit to a GP surgery (2 journeys – out and back) = 1.12 kgCO_2e

Emission factors source: SHC care pathway calculator [24]

7.2. Materials and activities required for study assessments that are in addition to standard of care

This includes everything that happens to the patient in the protocol schedule of assessments which is over and above routine care. This is not an exhaustive list; please inform us via cict-icrctsu@icr.ac.uk if your protocol specifies an activity that has not been included, and we will help to determine the associated carbon footprint.

Surgery

A 1-hour surgery = 53 kg CO_2e

A 30-minute surgery = 26.5 kg CO_2e

Emission factor source: SHC care pathway calculator [24]

Bed days

Low intensity (general ward) = 37.9 kg CO_2e

High intensity (ICU) = 103 kg CO_2e

Emission factor source: SHC care pathway calculator [24]

Scans

1 MRI = 24.7 kg CO_2e

Emission factor source: SHC care pathway calculator [24]

1 CT scan = 9.2 kg CO_2e

1 Chest X-Ray = 0.8 kgCO_2e

1 Ultrasound = 0.5 kgCO_2e

Emission factor source: The carbon footprint of hospital diagnostic imaging in Australia (thelancet.com) [25]

Radiotherapy

To calculate the carbon footprint of radiotherapy treatments, the total power (kWh) per course has been multiplied by the 2023 UK electricity emission factor below:

- Prostate Conventional (28 fractions): 38.34 kWh x 0.257 = **9.85 kgCO_2e**
- Prostate SBRT (5 fractions): 5.03 kWh x 0.257 = **1.3 kgCO_2e**

- Breast Hypofractionated (15 fractions): 16.63 kWh x 0.257 = **4.27 kgCO_2e**
- Breast Hypofractionated (5 fractions): 8.45 kWh x 0.257 = **2.17 kgCO_2e**

- Lung Conventional (30 fractions): 33.32 kWh x 0.257 = **8.56 kgCO_2e**
- Lung SBRT (5 fractions): 7.32 kWh x 0.257 = **1.88 kgCO_2e**

Benchmark data source: Estimating Carbon Dioxide Emissions and Direct Power Consumption of Linear Accelerator–Based External Beam Radiation Therapy (nih.gov)[26]

Electricity emission factor source: GOV.UK Greenhouse gas reporting, conversion factors 2023 [7]

7.2. Materials and activities required for study assessments (continued)

Consumables

For a trial appointment where consumables such as gloves are required, account for **0.30 kgCO_2e per patient per appointment.**

Emission factor source: SHC care pathway calculator guidance GP consultation module, 2015 [27]

Blood investigations

CO_2 e emissions for haematology tests:
- 82 g/test (95% CI, 73-91 g/test) for coagulation profile
- 116 g/test (95% CI, 101-135 g/test) for full blood examination.

CO_2 e emissions for biochemical tests:
- 0.5 g/test CO_2 e (95% CI, 0.4-0.6 g/test) for C-reactive protein (low because typically ordered with urea and electrolyte assessment)
- 49 g/test (95% CI, 45-53 g/test) for arterial blood gas assessment
- 99 g/test (95% CI, 84-113 g/test) for urea and electrolyte assessment.

NB: These emissions include the materials and consumables required for sample collection, phlebotomy and analysis, as well as power consumption by pathology analysers.

Emission factor source: The carbon footprint of pathology testing. Scott McAlister, Alexandra L Barratt, Katy JL Bell and Forbes McGain. Med J Aust 2020; 212 (8): 377-382.
Published online: 4 May 2020 **The carbon footprint of pathology testing - McAlister - 2020 - Medical Journal of Australia - Wiley Online Library** [28]

Other

A 30-minute phone call = 3g/0.003 kg CO_2e

Emission factor source: How Bad Are Bananas? Mike Berners-Lee [29]

Oxygen Gas (600g per cannister) = 0.24543 Kg CO_2e per cannister

Emission factor source: Ecoinvent, version 2.2, 2011 [6]

1 litre of saline = 0.1143197 kg CO_2e per litre

Emission factor source: Ecoinvent, version 2.2, 2011 [6]

A disposable dental examination kit (containing a mirror, probe and tweezers) = 0.302644 kg CO_2e per kit. Carbon footprint includes component manufacture and materials, sterilisation, packaging, transport and disposal.

Emission factor source: Byrne, D., Saget, S., Davidson, A. et al. Comparing the environmental impact of reusable and disposable dental examination kits: a life cycle assessment approach. Br Dent J 233, 317–325 (2022). https://doi.org/10.1038/s41415-022-4912-4 [30]

A dental examination = 5.50 kg CO_2e per examination. Carbon footprint includes staff and patient travel, procurement, energy and water usage and generic disposables used for all procedures.

Emission factor source: An estimated carbon footprint of NHS primary dental care within England. How can dentistry be more environmentally sustainable? (nature.com) [31]

7.3. Utilities required for study assessments that are in addition to standard of care

It can be difficult to calculate the carbon footprint associated with energy consumption by hospital staff directly because a hospital (and the equipment and staff within in it), are used for many other non-trial activities. Emissions are therefore estimated based on average per person emissions and the FTE of the trial hospital staff.

This is calculated by multiplying the average space occupied by a hospital staff member (16.5 m^2) by the kWh used per m^2 of a hospital (86 kWh/m^2). The kWh per FTE per year is then multiplied by the electricity emission factor provided below to calculate the carbon footprint attributed per hospital staff FTE. Finally, multiply by the number of years and FTE applicable**.** The calculation is exemplified below.

Calculation

- 16.5 m^2 x 86 kWh/m^2 = 1419 kWh per FTE per year

2023 UK electricity emission factor = 0.257 kg CO_2e per kWh

- 1419 kWh x 0.257 = **364.7 kgCO_2e per FTE per year**

Multiply 364.7 kgCO_2e by the hospital staff FTE required for the whole trial duration. Use the trial SOECAT or the costing included in the initial funding application (if predates use of SOECAT) to establish the FTE or total number of hours required by hospital staff for the trial. If using the number of hours, please follow the below method to establish the FTE required for the calculation.

Number of hours in SOECAT / 1762.5 = FTE required for trial.

Example calculation:

If 449.25 total hospital staff hours required for trial:

449.25 / 1762.5 = 0.25. Therefore 25% of 1 hospital staff FTE required.

364.7 kgCO_2e x 0.25 = 91.2 kgCO_2e

Assumption: The FTE of a nurse/hospital staff is 1762.5 hours. The standard full-time working week for NHS staff is 37.5 hours. 52 weeks x 37.5 = 1950 hours, minus 35 days/5 weeks a year off = 1762.5 hours.

Assumption: each health care professional occupies 16.5m^2 room, source HBN 12 (england.nhs.uk) page 32 [32]

Hospital benchmark data source: The Non-Domestic National Energy Efficiency Data-Framework 2023 (England and Wales) (publishing.service.gov.uk) [9]

Electricity emission factor source: Greenhouse gas reporting: conversion factors 2023, GOV.UK [7]

7.3. Utilities required for study assessments that are in addition to standard of care (continued)

Heating

For heating, the calculation follows the same method as above. The 16.5m^2 per person is multiplied by the health building heating benchmark and then by the natural gas conversion factor provided below.

2023 Health building heating benchmark: **188** (kWh/m^2)

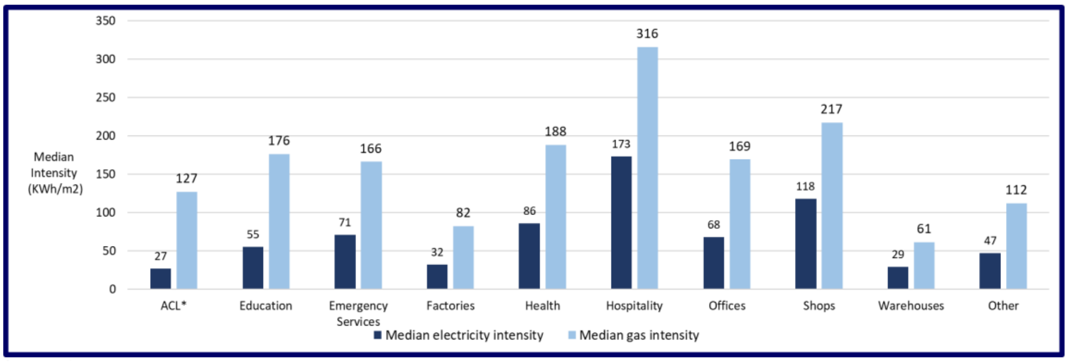

2023 UK Natural gas conversion factor: **0.213**

Calculation

16.5 x 188 = 3102 kWh per FTE per year
3102 x 0.213 = 660.7 kgCO_2e per FTE per year

Multiply 660.7 kgCO_2e by the hospital staff FTE required for the whole trial duration.

Assumption: each health care professional occupies 16.5m^2 room, source HBN 12 (england.nhs.uk) page 32 [32]

Assumption: If the heating source is unknown, assume that the heating source is natural gas.

Hospital benchmark data source: The Non-Domestic National Energy Efficiency Data-Framework 2023 (England and Wales) (publishing.service.gov.uk) [9]

Natural gas emission factor source: Greenhouse gas reporting: conversion factors 2023, GOV.UK [7]

8 Samples

This module includes the following activities:

8.1. Materials involved
8.2. Movement of sample kits from manufacturer to CTU
8.3. Movement of sample kits from CTU/distributor to participating sites
8.4. Movement of sample from participating sites to central laboratory

8.1. Materials involved in sample collection and distribution

The emissions attributed to sample collection consumables for common blood tests are included in the blood investigations section of 7.2.

Below is a list of commonly used materials used in sample collection and distribution and their equivalent emission factor. Multiply the kg of the material by the relevant emission factor provided below to determine the kg CO_2e.

- Paper: 0.91048 kg CO_2e per kg
- Cardboard: 0.8015 kg CO_2e per kg
- Plastics (average): 3.10245 kg CO_2e per kg
- Plastics (average plastic film): 2.56026 kg CO_2e per kg
- Plastics (average plastic rigid): 3.26392 kg CO_2e per kg
- Plastics (HDPE): 3.25593 kg CO_2e per kg
- Plastics (LDPE and LLDPE): 2.58673 kg CO_2e per kg
- Plastics (PET): 4.01848 kg CO_2e per kg
- Plastics (PP): 3.09082 kg CO_2e per kg
- Plastics (PS): 3.76404 kg CO_2e per kg
- Plastics (PVC): 3.39918 kg CO_2e per kg
- Glass: 1.40277 kg CO_2e per kg

Example: 100 x 10ml PET blood tubes (such as Streck), weight 5kg.
5kg x 4.032 (emission factor for PET) = 20.2 kgCO_2e

Example: 100 slide mailing containers made of polypropylene, weight 1.02 kg
1.02kg x 3.105 = 3.2 kgCO_2e

Example: sample mailing container made of polypropylene, 0.0145 kg per individual container e.g. Product - Sarstedt
0.0145 kg x 3.09082 = 0.045 kgCO_2e per container

Example: cardboard mailing box, 194 x 125 x 68mm, 0.07857 kg per box
0.07857 kg x 0.8015 = 0.063 kgCO_2e per box

Example: Styrofoam inner box for sample transport, 0.0371 kg per box.
0.0371 kg x 3.76404 = 0.14 kgCO_2e per box

Material emission factor sources: Greenhouse gas reporting: conversion factors 2023, GOV.UK[7]

For 8.2., 8.3., and 8.4, please refer to section 1.2. for freight and 4.1.1 for refrigerated/ frozen freight.

9 Laboratory

This module includes the following activities:

- **9.1. Emissions attributed to lab utilities according to staff FTE**
- **9.2. Materials/equipment/consumables used in processing and analysis of samples**
- **9.3. Storage of samples e.g., utilities and ultra-low temperature freezer**

9.1. Laboratory utilities according to staff FTE

Electricity

The carbon footprint associated with energy consumption by laboratory staff can be difficult to calculate directly because a laboratory, and the equipment and staff within in it, are used for many other non-trial activities. Emissions are therefore estimated based on average per person emissions and the FTE of the trial laboratory staff.

This is calculated by multiplying the average space occupied by a laboratory staff member (40 m^2) by the kWh used per m^2 of a laboratory (160 kWh/m^2). The kWh per FTE per year is then multiplied by the electricity emission factor provided below to calculate the carbon footprint attributed to 1 FTE for 1 year. Finally, multiply by the number of years and FTE applicable**.** The calculation is exemplified below. Use the trial SOECAT or the costing included in the initial funding application (if predates use of SOECAT) to establish the FTE or total number of hours required by laboratory staff.

Calculation

- 40m^2 x 160kWh = 6400 kWh per FTE per year
  2023 UK electricity emission factor = 0.257 kg CO_2e per kWh
- 6400 kWh x 0.257 = 1644.8 kgCO_2e per FTE per year

Multiply 1644.8 kgCO_2e by the laboratory staff FTE required for the whole trial duration.

Heating

For heating, the calculation follows the same method as above. The 40m^2 per person is multiplied by the laboratory heating benchmark and then by the natural gas conversion factor provided below:

- Laboratory fossil thermal typical benchmark: **160** kWh per year per sqm floor area (kWh/m^2)
- 2023 UK Natural gas conversion factor**: 0.213**

Calculation:

40m^2 x 160kWh = 6400 kWh per FTE per year

6400 kWh x 0.213 = 1363.2 kgCO_2e per FTE per year

Multiply 1363.2 kgCO_2e by the laboratory staff FTE required for the whole trial duration.

Assumption: For R&D, 40m^2 required per FTE according to UK EMPLOYMENT DENSITY GUIDE, 3rd edition November 2015 [8]
Assumption**:** If the heating source is unknown, assume that the heating source is natural gas.

Laboratory benchmark data source: Health Technical Memorandum 07-02: EnCO2de 2015 – making energy work in healthcare (england.nhs.uk) [33]
Emission factor source**:** Greenhouse gas reporting: conversion factors 2023, GOV.UK [7]

9.2. Materials/equipment/consumables used in processing and analysis of samples

To avoid double counting, use of equipment will be included in lab staff FTE if calculated.

However, if the trial does not involve a central lab, but there is still sample processing on site, please see below. For storage of samples, please see section 9.3.

To calculate the emissions of a piece of equipment, multiply the power consumption in Watts by hours used to get a kWh value. Depending on the equipment, this can often be found in the specifications of a listed product. Finally multiply kWh by the electricity emission factor (0.257).

Example: use of a 310-Watt centrifuge for 15 minutes.

310 Watts x 0.25 (hours) = 77.5 kWh

- 77.5 kWh x 0.257 = 19.9 kg CO_2e

Consider the centrifuge capacity and multiply by the number of uses required.

Electricity emission factor source: Greenhouse gas reporting: conversion factors 2023, GOV.UK [7]

9.3. Storage and destruction of biological samples

For storage of samples in a fridge/-20 freezer or an ultra-low temperature freezer, the kWh usage per day is multiplied by 365 to calculate the kWh per year. This is then multiplied by the electricity emission factor and the number of years the samples are stored.

2023 UK electricity emission factor = 0.257 kg CO_2e per kWh

Fridge/-20 freezer

- 3kWh/day x 365 days = 1095 kWh per year
- 1095 x 0.257 = 281.4 kgCO_2e per year
- Multiply by number of years stored

-80 freezer

- 22kWh/day x 365 days = 8030 kWh per year
- 8030 x 0.257 = 2063.7 kgCO_2e per year
- Multiply by number of years stored

NB: The kgCO_2e above are for the whole fridge/freezer for 1 year - **you will need to make an assumption about the amount of space in the freezer that the trial samples take up.** As a guide, a typical ULT freezer at full capacity will store 50,000 microtubes.

Example: if the samples take up a third of the freezer space

2063.7 kg CO_2e x 0.333 = 687.2 kg CO_2e

Assumptions: A -80°C freezer uses 22 kWh/day, a -20°C freezer uses 3 kWh/day **(**Source: Did You Know? - International Laboratory Freezer Challenge) [34]

Electricity emission factor source: Greenhouse gas reporting: conversion factors 2023, GOV.UK [7]

10 Trial close out

This module includes the following activities:

10.1. Storage and archiving of essential trial documentation and data
10.2. Storage and destruction of biological samples
10.3. Return of equipment and supplies from participating sites to CTU

10.1. Storage and archiving of essential trial documentation and data

For the storage of archived documentation, estimate the m^2 required for archiving. This estimate will then be multiplied by the most suitable energy benchmark from the list provided below. Choose the type of building most similar to where the documents are stored in e.g. office/lab/warehouse/health building. Finally multiply by the electricity emission factor (0.257).

Energy benchmarks:

- office = 68 kWh/m^2
- laboratory = 160 kWh/m^2
- warehouse = 29 kWh/m^2
- health building = 86 kWh/m^2

Calculation

- m^2 required x benchmark = kWh
- kWh x 0.257 = kg CO_2e for 1 year of storage
- Multiply by number of years necessary.

NB: Approximately 12 archive boxes fit inside $1m^2$.

Heating

For heating, use the same method as above. Estimate the m^2 used and multiply by the corresponding heating benchmark (i.e., if 'laboratory' was used above then select the same for this calculation). Finally multiply by the UK natural gas emission factor (0.213).

Benchmarks:

- office = 169 kWh/m^2
- laboratory = 160 kWh/m^2
- warehouse = 61 kWh/m^2
- health building = 188 kWh/m^2

Calculation

- m^2 required x benchmark = kWh
- kWh x 0.213 = kgCO_2e for 1 year of storage
- Multiply by number of years necessary.

Assumption: If the heating source is unknown, assume heating source is natural gas.

Benchmark data source: The Non-Domestic National Energy Efficiency Data-Framework 2023 (England and Wales) (publishing.service.gov.uk) [9]

Electricity and natural gas emission factor source: Greenhouse gas reporting: conversion factors 2023, GOV.UK [7]

10.1. Storage and archiving of essential trial documentation and data (continued)

For electronic data or documentation storage, estimate 1.285 $kgCO_2e$ per GB per year.

Emission factor source: Costenaro, D. and Duer, A. (n.d.). The Megawatts behind Your Megabytes: Going from Data-Center to Desktop. [18]

10.2. Storage and destruction of biological samples

See section 9.3. for storage of refrigerated or frozen samples.

See section 10.1 for storage of ambient samples.

10.3. Return of equipment and supplies from participating sites to CTU

See section 1.2. for freight.

References

[1] Sustainable Trials Study Group. Towards sustainable clinical trials. *BMJ* 2007;334:671
[2] Lyle K, Dent L, Bailey S, et al. Carbon cost of pragmatic randomised controlled trials: retrospective analysis of sample of trials. *BMJ* 2009;339:b4187
[3] Making clinical trials sustainable [Internet]. The Sustainable Healthcare Coalition. [cited 2023 May 11]. Available from: https://shcoalition.org/clinical-trials/
[4] Climate change and health [Internet]. World Health Organization. 2021 October 30 [cited 2023 May 11]. Available from: https://www.who.int/news-room/fact-sheets/detail/climate-change-and-health
[5] What is an emission factor? [Internet]. Climfoot-project.eu. [cited 2023 May 11]. Available from: https://climfoot-project.eu/en/what-emission-factor
[6] Ecoinvent, version 2.2. 2011. Available from: https://ecoinvent.org/the-ecoinvent-database/data-releases/ecoinvent-version-2/
[7] Greenhouse gas reporting: conversion factors [Internet]. GOV.UK 2023. [cited January 2024]. Available from: https://www.gov.uk/government/publications/greenhouse-gas-reporting-conversion-factors-2023
[8] EMPLOYMT DENSITY GUIDE (3rd Edition) [Internet]. Homes & Communities Agency. November 2015 [cited 2023 May 11]. Available from: https://www.kirklees.gov.uk/beta/planning-policy/pdf/examination/national-evidence/NE48_employment_density_guide_3rd_edition.pdf
[9] The Non-Domestic National Energy Efficiency Data-Framework (England and Wales) [Internet]. Department for Business, Energy & Industrial Strategy. 2023 [cited January 2024]. Available from: https://assets.publishing.service.gov.uk/media/64e62d47db1c07000d22b345/nd-need-2023-report.pdf
[10] Transport Statistics Great Britain: 2022 [Internet]. GOV.UK (www.gov.uk). 2023 December 14 [cited January 5 2024]. Available from: https://www.gov.uk/government/statistics/transport-statistics-great-britain-2023/transport-statistics-great-britain-2022-domestic-travel
[11] Commuting trends in England 1988-2015 [Internet]. GOV.UK. 2017 November 7 [cited 2023 May 11]. Available from: https://www.gov.uk/government/publications/commuting-trends-in-england-1988-to-2015
[12] Kayla Wiles. Turn off that camera during virtual meetings, environmental study says [Internet]. Purdue University News. 2021 January 14 [cited 2023 May 11]. Available from: https://www.purdue.edu/newsroom/releases/2021/Q1/turn-off-that-camera-during-virtual-meetings,-environmental-study-says.html
[13] Gov.UK Government conversion factors for company reporting of greenhouse gas emissions 2012 – Annex 13 (with consideration of 2020 inflation rates)
[14] WWF. 2018. Food in a warming world [Internet]. [cited 2023 May 11]. Available from: https://www.wwf.org.uk/sites/default/files/2018-03/Food_in_a_warming_world_report.PDF?source=post_page---------------------------
[15] Goellner et al. The International Journal of Life Cycle Assessment. Vol 19, pp 611-619 (2014)
[16] Weber et al. Journal of Industrial Ecology, "The Energy and Climate Change Impacts Of Different Music Delivery Methods". Vol 14, Issue 5, pg. 754-769 (2010)
[17] What's the carbon footprint of an email? [Internet]. Mail.com blog. 202 April 21 [cited 2023 May 11]. Available from: https://www.mail.com/blog/posts/email-carbon-footprint/9/
[18] Costenaro, D. and Duer, A. The Megawatts behind Your Megabytes: Going from Data-Center to Desktop. [online] Available at: https://www.aceee.org/files/proceedings/2012/data/papers/0193-000409.pdf. (cited 2024 July 30)
[19] Srilatha Manne. Examining the Carbon Footprint of Devices [Internet]. Microsoft.com. 2020 November 23 [cited 2023 May 11. Available from: https://devblogs.microsoft.com/sustainable-software/examining-the-carbon-footprint-of-devices/
[20] Obringer, R., Rachunok, B., Maia-Silva, D., Arbabzadeh, M., Nateghi, R., & Madani, K. (2021). The overlooked environmental footprint of increasing Internet use. *Resources, Conservation and Recycling*, *167*, [105389]. https://doi.org/10.1016/j.resconrec.2020.105389
[21] What Is The Carbon Footprint Of A Laptop? [Internet]. Circular Computing™. 2021 August 9 [cited 2024 January 5]. Available from: https://circularcomputing.com/news/carbon-footprint-laptop/
[22] Apple watch SE. Product Environmental Report – Apple [Internet]. [cited 2023 May 11]. Available from: https://www.apple.com/by/environment/pdf/products/watch/Apple_Watch_SE_PER_sept2020.pdf
[23] Meinrenken, C.J., Chen, D., Esparza, R.A. et al. The Carbon Catalogue, carbon footprints of 866 commercial products from 8 industry sectors and 5 continents. Sci Data 9, 87 (2022). https://doi.org/10.1038/s41597-022-01178-9. Catalogue available from: https://springernature.figshare.com/articles/dataset/The_Carbon_Catalogue_public_database_Carbon_footprints_of_866_commercial_products_across_8_industry_sectors_and_5_continents/16908979
[24] Care pathways carbon footprint calculator [Internet]. Sustainable Healthcare Coalition. [cited 2023 May 11]. Available from: https://shcpathways.org/full-calculator/
[25] McAlister S, McGain F, Petersen M, Story D, Charlesworth K, Ison G, Barratt A. The carbon footprint of hospital diagnostic imaging in Australia. Lancet Reg Health West Pac. 2022 May 3;24:100459. doi: 10.1016/j.lanwpc.2022.100459. PMID: 35538935; PMCID: PMC9079346.
[26] Shenker RF, Johnson TL, Ribeiro M, Rodrigues A, Chino J. Estimating Carbon Dioxide Emissions and Direct Power Consumption of Linear Accelerator-Based External Beam Radiation Therapy. Adv Radiat Oncol. 2022 Dec 31;8(3):101170. doi: 10.1016/j.adro.2022.101170. PMID: 36798606; PMCID: PMC9926191.

[27] SHC care pathway calculator guidance, GP consultation module, page 18, 2015. Available from: https://shcoalition.org/sustainable-care-pathways-guidance/
[28] McAlister, S., Barratt, A.L., Bell, K.J. and McGain, F. (2020), The carbon footprint of pathology testing. Med. J. Aust., 212: 377-382. https://doi.org/10.5694/mja2.50583
[29] Berners-Lee, M. (2010). How bad are bananas? The carbon footprint of everything.
[30] Byrne, D., Saget, S., Davidson, A. et al. Comparing the environmental impact of reusable and disposable dental examination kits: a life cycle assessment approach. British Dental Journal 233, 317–325 (2022). https://doi.org/10.1038/s41415-022-4912-4
[31] Duane, B., Lee, M., White, S. et al. An estimated carbon footprint of NHS primary dental care within England. How can dentistry be more environmentally sustainable? British Dental Journal, 223, 589–593 (2017). https://doi.org/10.1038/sj.bdj.2017.839
[32] Health Building Note (HBN) 12, page 32. NHS Estates. 2004 [cited 2023 May 11]. Available from: https://www.england.nhs.uk/wp-content/uploads/2021/05/HBN_12.pdf
[33] Health Technical Memorandum 07-02: EnCO2de 2015 – making energy work in healthcare. Department of Health. 2015 [cited 2023 May 11]. Available from: https://www.england.nhs.uk/wp-content/uploads/2021/05/HTM_07-02_Part_A_FINAL.pdf
[34] Freezer challenge blog. Did you know? [Internet]. Freezerchallenge.org. 2020 June 19 [cited 2023 May 11]. Available from: https://www.freezerchallenge.org/fc-blog/did-you-know

Acknowledgements

This work was produced through funding from the NIHR entitled **NIHR Clinical Trials Unit Support Funding Opportunity – Supporting efficient / innovative delivery of NIHR research**

Authors: Jessica Griffiths, Lisa Fox, Paula Williamson

On behalf of the Low Carbon Clinical Trials working group: Fiona Adshead, Rustam Al-Shahi Salman, Craig Anderson, Emma Bedson, Judith Bliss, Ana Boshoff, Xiaoying Chen, Denise Cranley, Peter Doran, Carrol Gamble, Kerenza Hood, Naomi McGregor, Carolyn McNamara, Elis Midha, Keith Moore, Alexis M Perkins, Sarah Pett, Matthew R Sydes.

Grateful thanks to Environmental Resources Management for their technical support and expertise in the development of the method.

Table of changes

Version	Date	Type of amendment	Description of change
V0.2	14.02.2023	Admin changes to entire document	Changed 'patient' to 'participant' everywhere except trial specific patient assessments.<br><br>Changed 'Sponsor' to 'CTU'<br><br>Reordered 'Intervention' section.<br><br>Clarified that 'email traffic' is 'an estimate of all emails exchanged between CTU and participating sites throughout the study lifetime, including data query resolution emails.<br><br>Added 'in addition to standard of care' to 7.2. and 7.3 heading.<br><br>Clarified consumables are 'per patient per appointment'<br><br>Renamed section 10 'Analysis and trial close out' and added statement 'NB: Analysis does not need to be calculated separately, it is covered by the emissions attributed to trial staff FTE in "CTU emissions" and "Data Collection and exchange".'
		Emission factor added to Section 4, Intervention	Added the emission factor for polystyrene.
V0.3	31.03.2023	Emission factor added to Section 2.2, CTU emissions<br><br>Emission factor updated in Section 5.1, Data collection and exchange	Emission factor for homeworking added.<br><br>Emission factor for electronic file storage changed.
V0.4	25.05.2023	Admin change to Assumptions	Additional guidance provided around calculating activities in addition to routine care

V0.5	16.01.2024	Emission factors added and updated throughout entire document.	Existing emission factors have been updated in line with 2023 data from GOV.UK. Calculations using electricity and natural gas emission factors were updated, along with freight, business travel, building energy benchmarks and other clinical activities e.g. radiotherapy.<br><br>Emission factors for blood pressure monitoring, saline use, oxygen use, business travel by car, commuting using activity data, dental examinations, laptop usage and telephony added.<br><br>Additional assumptions have been included to aid the user with the calculations, for example the number of samples that can be stored in a freezer, the number of working hours in one full time equivalent (FTE), the number of folders that can be stored in $1m^2$ and the carbon footprint of common sample kit supplies.
