# Supplementary material for: What is the carbon footprint of academic clinical trials? A study of hotspots in 10 trials
Source: BMJ Open. 2024 Oct 16;14(10):e088600. doi: 10.1136/bmjopen-2024-088600 (PMC11487931; doi:10.1136/bmjopen-2024-088600)
Supplement: online supplemental file 3 [file bmjopen-14-10-s003.pdf]

FUNDED BY NIHR | National Institute for Health and Care Research

LIVERPOOL CLINICAL TRIALS CENTRE LCTC

ICR

Enabling lower carbon clinical trials: A method to quantify the carbon footprint of clinical trials to inform future lower carbon trial design

Guidance and method to calculate the carbon footprint of a clinical trial

Data collation quick guide and worksheet

This guidance provides information on how to carbon footprint a clinical trial for the purposes of the NIHR-funded project 'enabling lower carbon clinical trials.'

Within the guidance, clinical trial processes have been sub-divided into the following modules:

1. Trial set up
2. CTU emissions
3. Trial specific meetings and travel
4. Treatment intervention
5. Data collection and exchange
6. Trial supplies and equipment
7. Trial specific patient assessments
8. Samples
9. Laboratory
10. Trial close out

This list is not exhaustive, and it is expected that further activities and modules may need to be added to account for specialist processes in all clinical trial types.

NB: analysis of data does not need to be calculated separately, it is covered by the emissions attributed to trial staff FTE in "CTU emissions" and calculations included within "Data Collection and exchange".

In addition to this quick guide and worksheet, we have produced a detailed guidance and method document defining the project scope, limitations and assumptions. The detailed guidance contains a more in depth look and explanation of the calculations found in this document, including emission factor and benchmark data sources, and should be referred to when using this worksheet.

Introduction to calculating carbon footprint

A carbon footprint is a measure of greenhouse gases, usually quoted in kg or tonnes of carbon dioxide equivalent (CO_2e). To calculate the carbon footprint of a particular clinical trial process, both ‘activity data’ and ‘emission factors’ are required.

An emission factor, also known as a conversion factor, “is a coefficient which allows you to convert activity data into greenhouse gas emissions. It is the average **emission** rate of a given source, relative to units of activity or process/processes.”[1]

The activity data is provided by the user and multiplied by the emission factors provided in this guidance document.

Data collation quick guide and worksheet

This data collation quick guide should be used in conjunction with the “Enabling lower carbon clinical trials: A method to quantify the carbon footprint of clinical trials to inform future lower carbon trial design - Detailed Guidance and method to calculate the carbon footprint of a clinical trial”. The guidance document provides the detailed explanation of how calculations should be considered and calculated. This quick guide should be used to collate the trial-specific processes, necessary activity data and to record the subsequent calculations. It is important to avoid double-counting activities i.e., modules must not include activities already covered elsewhere in the clinical trial process map. Please complete this worksheet for each trial to be carbon footprinted.

NB: We are using the term ‘CTU’ to describe the organisation that manages all aspects of central trial management. For some institutions some of those tasks maybe done by groups outside the CTU team e.g., sponsor office/CRO etc.

Module	Examples	Trial activity data	Calculation	Total
**1. Trial set-up**				
1.1. Production of trial documentation to be sent to sites or participants	E.g. Site Investigator File and contents, Site Pharmacy File and contents, CRF Folder and contents, PIS/Cs, GP letters	Number of pages:<br><br>Number of folders used to send trial documentation:<br><br>**For eTMF:**<br>GB required for data storage and transmission:<br>Duration of data storage:	**Paper:**<br>[no. of page] x 0.005 = paper weight (kg)<br><br>b/w printing: Kg of paper x 0.22438 = kg CO_2e<br>Colour printing: Kg of paper x 0.31786 = kg CO_2e<br><br>Materials (paper): Kg of paper x 0.91048 = kg CO_2e<br><br>**Folders**:<br>Kg (of cardboard) x 0.8015 = kg CO_2e<br>Assumption: Weight of lever arch = 0.5kg<br>Assumption: Weight of ring binder = 0.3kg<br><br>**eTMF:**<br>Estimate 1.285 kg CO_2e per GB per year.	

1.2. Provision/postage of trial documentation to sites<br><br>1.3. Provision/postage of documentation to participants by CTU or participating sites<br><br>1.4. Provision/postage of incentives to participant	E.g. Site Investigator File and contents, Site Pharmacy File and contents, CRF Folder and contents, PIS/Cs, GP letters	Estimated weight of delivery (road):<br><br>Estimated distance of delivery (road):<br><br>Estimated weight of delivery (air):<br><br>Estimated distance of delivery (air):	Delivery weight (tonnes) x distance (km) = t.km<br><br>For road freight: t.km x 0.19443 = kg CO_2e<br><br>For air freight: t.km x required emission factor below = kg CO_2e<br><br>- Domestic (to/from UK) = 5.247686<br>- Short-haul (to/from UK) = 1.873305<br>- Long-haul (to/from UK) = 1.234192<br>- International (to/from non-UK) = 1.234192<br><br>NB: For delivery of trial supplies to patients or GP, if unknown, use 17.4km as distance from hospital to patient, or hospital to GP.	
**2. CTU emissions**				
2.1. Energy consumption at CTU according to trial staff FTE	E.g. energy consumption per square metre of air-conditioned office space	Staff FTE required for the whole trial duration:	**Energy consumption for 1 FTE for 1 year** = 209.7 kgCO_2e<br><br>Multiply by the CTU staff FTE required for the whole trial duration.	

2.2. Heating	E.g. energy consumption at coordination centre attributed to heating (natural gas), homeworking	Staff FTE required for the whole trial duration:	**Heating for 1 FTE for 1 year =** 432 kgCO_2e<br><br>Multiply by the CTU staff FTE required for the whole trial duration.<br><br>**Homeworking =** 0.33378 kgCO_2e per FTE working hour (includes electricity for office equipment and heating).<br><br>Total FTE working hours x 0.33378 = kgCO_2e<br><br>NB: You may assume 1 FTE is equal to 1800 hours.	
2.3. Trial team commuting	E.g. Car, rail, bus, walking etc	For calculation of commuting emissions using primary data you will need to know:<br>- Mode of transport<br>- Distance travelled<br><br>If primary data is unavailable, average commuting emissions are multiplied by:<br>- Trial staff FTE<br>- Trial Duration	For commuting by car or motorbike:<br><br>Distance travelled by vehicle (km) x required emission factor below = kg CO_2e<br>- Average petrol car = 0.209419 kg CO_2e per km<br>- Average diesel car = 0.211276 kg CO_2e per km<br>- Average hybrid car = 0.150069 kg CO_2e per km<br>- Average motorbike = 0.143234 kg CO_2e per km	

			For commuting by public transport:<br><br>Km travelled by passenger x required emission factor below = kg CO_2e<br>- National rail: 0.044433 kg CO_2e per passenger.km<br>- London Underground: 0.035082 kg CO_2e per passenger.km<br>- Light rail/tram: 0.036093 kg CO_2e per passenger.km<br>- Local bus (not London): 0.147233 kg CO_2e per passenger.km<br>- London Bus: 0.097483 kg CO_2e per passenger.km<br><br>If primary data is unavailable:<br><br>Total emissions attributed to 1 FTE commuting for 1 year = 987.7 kg CO_2e<br><br>**Multiply by the number of years and FTE applicable**	
**3. Trial specific meetings and travel**				
3.1. Visits and travel to site	E.g. Feasibility, site	Estimated distance travelled:	For business travel by car or public	

3.2. Travel to meetings	initiation and monitoring visits, audits, inspections, TMG, TSC, IDMC, and investigator meetings, PPIE, conferences, scientific meetings etc	Number of passengers:<br>Mode of transport:	transport please refer to the emission factors above in 2.3<br><br>For international rail:<br>Total distance travelled by passenger (km) x 0.005629 = kg CO_2e<br><br>For flights:<br>Total distance travelled by passenger (km) x relevant emission factor below:<br>- Domestic (average) to/from UK: 0.30608<br>- Short-haul (average) to/from UK: 0.20878<br>- Long-haul (average) to/from UK: 0.29341<br>- International (average) to/from non-UK: 0.19742<br><br>NB: Distances may be calculated using google maps and calculated from CTU to destination<br><br>**Videoconferencing** = 0.1573 kg CO_2e per person per hour	
3.3. Hotel stays	E.g. monitoring visits, audits, inspections etc	Number of rooms:<br>Number of nights:	For UK: number of hotel rooms x number of nights x 10.4 = kgCO_2e	

			For UK (London) = number of rooms x number of nights x 11.5 = $kgCO_2e$.<br><br>For other countries use conversion factors from orange 'hotel stay' tab: [ghg-conversion-factors-2023-full-file-update.xlsx (live.com)]<br><br>Alternatively, you may use cost-based method:<br>£ spent x 0.358 = $kgCO_2e$	
3.4. Sustenance	E.g. meeting lunches, hotel dinners	Number of lunches or dinners:	Meeting lunches or hotel dinners (vegetarian) = 2.6 $kgCO_2e$ per meal per person<br><br>Meeting lunches or hotel dinners (meat) = 5.92 $kgCO_2e$ per meal per person	
**4. Intervention*** **4.1. Physical (IMP)**<br>**4.2. Clinical (non-IMP)**<br>**4.3. Other (not captured above)**<br>**Please fill out the section most relevant to the intervention being investigated.**				
**4.1. Physical**				
4.1.1, 4.1.2. Movement of	E.g. movement of	Estimated weight and distance	Carry out freight calculation as	

* As per assumptions detailed in the guidance, manufacture of the intervention is considered out of scope. This section defines all processes relating to providing and delivering the trial intervention that are over and above routine care.

intervention, or materials required to deliver the intervention	intervention from manufacturing site to distribution site, shipment of IMP to participating sites or direct to participant	of delivery (t.km):	described in section 1.2.<br><br>For **refrigerated freight**, increase the total kgCO_2e associated with freight by 15%.<br><br>**Frozen freight:**<br>Dry ice has a carbon footprint of **1.81kg CO_2e for 1 kg dry ice** produced/used.<br><br>When calculating the overall emissions of frozen freight, as well as the 1.81 kgCO_2e per 1kg attributed to manufacture, include the weight (kg) of dry ice used in the weight of the freight calculation in section 1.2. In the absence of activity data, assume 1kg of dry ice is used per individual sample shipping box.<br><br>**For sea freight:**<br>1. Delivery weight (tonnes) x distance (km) = t.km<br>2. T.km x relevant emission factor below = kg CO_2e<br>- Emission factor for freight via 'average container ship' = 0.01977 kg CO_2e per t.km	

			- Emission factor for freight via 'average RoRo-Ferry' = 0.06328 kg CO_2e per t.km<br>-<br>**NB:** A RoRo-Ferry is a ship which allows easy loading and disembarking of vehicles carrying freight.	
4.1.3. Materials required for the packaging and shipment of IMP	E.g. cardboard, cold storage boxes, polystyrene	Number of cold storage boxes:<br><br>Kg of cardboard/ polystyrene:	Single use sample cold storage box = 25.2 kgCO_2e per box<br><br>Reusable sample cold storage box = 2.2 kgCO_2e per box<br><br>Kg (cardboard) x 0.8015 = kgCO_2e<br>Kg (polystyrene) x 3.76 = kgCO_2e	
4.1.4. Activities or resources required/relating to delivery of the intervention	E.g. low intensity bed days, pharmacy release, IMP preparation	FTE required by pharmacy/hospital staff for delivery of intervention:	Please refer to section 7.3 to calculate the emissions attributed to hospital or pharmacy staff time.<br><br>Please refer to section 7.2 for activities that may be relevant to the delivery of the intervention, e.g. a low intensity bed day.<br><br>For the carbon footprint of materials (e.g. plastic, paper, glass), please refer to section 8.1.	
4.1.5. Destruction of overage	E.g. incineration of IMP	Estimated weight of overage	Kg of waste x 2.4252 = kgCO_2e	

		incinerated:		
**4.2. Clinical** e.g., radiotherapy, device, surgical.<br>NB: not all calculations will be relevant to all interventions. **This section of the method will be further developed as we carbon footprint more trials, so please inform us via CICT-icrctsu@icr.ac.uk if your protocol specifies an activity that has not been included, and we will help to determine the associated carbon footprint.**				
4.2.1 Movement of the intervention, or resources required to deliver the intervention	E.g. movement of intervention from manufacturing site to distribution site, shipment of intervention to participating sites.	Estimated weight and distance of delivery (t.km):	Please refer to section 1.2 and 4.1.1, 4.1.2.	
4.2.2 Materials required for the packaging and shipment of the intervention	E.g. cardboard, cold storage boxes, polystyrene	Number of cold storage boxes:<br><br>Kg of cardboard/ polystyrene:	Please refer to section 4.1.3.	
4.2.3 Utilities required for delivery of the intervention	E.g. Hospital utilities if the intervention is delivered within a hospital	Hospital staff FTE required:	Please refer to section 7.3 to calculate the emissions attributed to hospital utilities if required to deliver the intervention.	

4.2.4 Activities or resources required/relating to delivery of the intervention	E.g. consumables, surgical time, specialist equipment, incineration of surgical waste		Please refer to section 7.2 for consumables, surgery and other activities that may be relevant to the delivery of the intervention, but please take care to avoid double counting.<br><br>To calculate the emissions attributed to incineration, e.g. of surgical waste, please refer to section 4.1.4.	
**4.3. Other**<br>NB: not all calculations will be relevant to all interventions. **This section of the method will be further developed as we carbon footprint more trials, so please inform us via CICT-icrctsu@icr.ac.uk if your protocol specifies an activity that has not been included, and we will help to determine the associated carbon footprint.**				
4.3.1 Movement of the intervention to the participant or participating site	E.g. shipment of intervention to participating sites or direct to participant	Estimated weight and distance of delivery (t.km):	Please refer to section 1.2.	
4.3.2 Materials required for packaging and shipment of the intervention	E.g. cardboard, cold storage boxes, polystyrene	Number of cold storage boxes:<br><br>Kg of cardboard/ polystyrene:	Please refer to section 4.1.3.	
4.3.3 Materials or resources required for delivery of the intervention	E.g. software, booklets, specialist equipment		For printing and paper, please refer to section 1.1.	
4.3.4 Travel required to facilitate delivery of the intervention	E.g. to deliver training, conduct interviews etc	Estimated distance travelled, number of passengers (p.km):	Please refer to section 3.1, 3.2.	
**5. Data collection and exchange**				

NB: analysis of data does not need to be calculated separately, it is covered by the emissions attributed to trial staff FTE in "CTU emissions" and calculations included within "Data Collection and exchange".				
5.1. Data collection and query exchange between CTU and sites	E.g. CRFs, EDC completion and query resolution, scans copied to CDs	Estimated weight and distance of deliveries (t.km):<br><br>Number of CDs and time taken to copy scans:<br><br>Number of emails:<br>NB: this is an estimate of all emails exchanged between CTU and participating sites throughout the study lifetime, including data query resolution emails.<br><br>GB required for data storage and transmission:<br>Duration of data storage:<br><br>NB: Web-based data entry at sites, e.g. CRF completion, will be accounted for in the time a hospital worker spends on the trial and the carbon footprint of the trial databases.	For postage of materials, please refer to section 1.2 (freight).<br><br>The carbon footprint of manufacturing a CD = 0.83 kg CO_2e per CD<br><br>The carbon footprint of copying the scans on to a CD using a computer = 0.18079 kg CO_2e per hour<br><br>An email without an attachment = 10g CO_2e. Double this for an email with a one-megabyte attachment.<br><br>Data storage and transmission: estimate 1.285 kg CO_2e per GB per year.	
5.2. Data sent direct from participants to CTU or participating sites	E.g. Questionnaires, patient diaries, wearables	Estimated weight, and distance of delivery (t.km):<br><br>Device used and time taken to complete electronic questionnaires:	For paper questionnaires, please refer to section 1.1. for the carbon footprint of producing the materials and section 1.2. for postage (freight).	

			For use of smart watches and other devices see section 6.3.<br><br>**Electronic questionnaires**<br>Add the emissions attributed to data storage and transmission to the emissions attributed to using a device to complete the questionnaire.<br><br>Web surfing (data storage and transmission) = 9.441 g CO2e/hr (10 mins = 1.57 g CO_2e)<br><br>Choose from the below:<br>- Desktop computer = 0.18079 kg CO_2e per hour<br>- Laptop = 0.028719 kg CO_2e per hour<br>- Tablet = 0.027397 kg CO_2e per hour<br>- Smartphone = 0.015068 kg CO_2e per hour	

5.3. Data from labs to CTU<br>5.4. Data from other collaborators to CTU	E.g. Laboratory patient results, data linkage	GB required for data storage and transmission:<br>Duration of data storage:<br><br>Total £ spent on computer services:	For electronic storage, estimate 1.285 $kgCO_2e$ per GB per year.<br><br>For computer services such as data linkage:<br>£ x 0.149 = $kgCO_2e$	
**6. Trial supplies and equipment**				
6.1. Equipment used by CTU	E.g. computers, laptops, printers, software	Total £ spent on office machinery and computers for trial:	A laptop = 422.5 $kgCO_2e$<br><br>For any other office machinery and computers purchased specifically for trial:<br>£ x 0.387 = $kgCO_2e$	
6.2. Equipment and supplies used by participating sites supplied by CTU	E.g. centrifuge, fridge, freezer	Estimated weight and distance of delivery(t.km):	For the shipment of equipment to participating sites, please refer to section 1.2.	
6.3. Equipment and supplies provided to participants specifically for the trial	E.g. wearables, smartphone, tablet	Number of devices and duration of their usage:<br><br>Estimated weight and distance of deliveries (t.km):	**Smartphone =** 55 $kgCO_2e$ from manufacture and add 5.5 $kgCO_2e$ per year of usage.<br><br>**Tablet =** 119 $kgCO_2e$ from manufacture and add 10 $kgCO_2e$ per year of usage.<br><br>**Wearables/smart watch** = 30.1 kg CO_2e for manufacture and add 1.633 kg CO_2e per year of usage.	

			**Upper arm automatic blood pressure monitor** = 28.2 kg CO_2e.<br>NB: considering a 3-year product lifetime, make sure to attribute emissions based on usage specifically for the trial.<br><br>**To calculate the carbon footprint associated with shipment of the devices, please refer to section 1.2.**	
**7. Trial specific patient assessments**				
7.1. Patient travel for study visits that are in addition to standard of care	E.g. Eligibility and screening assessments, trial-specific assessments and procedures	Number of times patient is required to travel (in addition to standard of care):<br><br>Number of patients:	If primary data (mode of transport and distance travelled) is available, please refer to section 3.1. Otherwise:<br><br>- Emissions associated with one patient visit to hospital (UK) = 5.8 kgCO_2e (this includes both the out and back journeys)<br><br>- Emissions associated with one patient visit to GP surgery (UK) = 1.12 kgCO_2e (this includes	

			both the out and back journeys)	
7.2. Materials and activities required for study assessments that are in addition to standard of care	E.g. Laboratory tests, imaging assessments, clinical activities relating to intervention for example administering of study drug, biopsy.	Patient schedule of assessments:	The carbon footprints of common patient activities or resources are provided below, but please refer to the detailed guidance for a full list of activities.<br><br>Consumables = 0.30 kg CO_2e per patient per trial appointment where consumables (such as gloves) required.<br><br>▪ 1 MRI = 24.7 kg CO_2e<br>▪ 1 CT scan = 9.2 kg CO_2e<br>▪ 1 Chest X-Ray = 0.8 kg CO_2e<br>▪ 1 Ultrasound = 0.5 kg CO_2e<br>▪ 1 hour in surgery = 53 kg CO_2e<br>▪ 1 low intensity (general ward) bed day = 37.9 kg CO_2e<br>▪ 1 high intensity (ICU) bed day = 103 kg CO_2e<br><br>Blood tests:<br>▪ 82 g CO_2e for	

			▪ coagulation profile<br>▪ 116 g CO_2e for full blood examination<br>▪ 49 g CO_2e for arterial gas assessment<br>▪ 99 g CO_2e for urea and electrolyte assessment<br>▪ 0.5 g CO_2e for C-reactive protein<br><br>**Please note that the above figures for blood tests include the materials and consumables required for sample collection, phlebotomy and analysis, as well as power consumption by pathology analysers.**	
7.3. Utilities required for study assessments that are in addition to standard of care	E.g. energy consumption per square metre of hospital space according to trial staff FTE, taking into account time required for CRF completion and study assessments, consent etc	Hospital staff FTE required for the whole trial duration:	**Electricity:**<br>1 FTE 1 year = 364.7 kg CO_2e<br><br>Multiply by the hospital staff FTE required for the whole trial duration.<br><br>**Heating:**<br>1 FTE, 1 year = 660.7 kg CO_2e<br><br>Multiply by the hospital staff FTE required for the whole trial duration.	

**8. Samples**				
8.1. Materials involved	E.g. sample collection kit and packaging for shipment	Kg of material:	The emissions attributed to sample collection consumables for common blood tests are included in the blood test carbon footprints listed in section 7.2.<br><br>The carbon footprint of other common materials:<br>- Average plastics: 3.10245 kg CO_2e per kg<br>- Plastics (average film): 2.56026 kg CO_2e per kg<br>- Plastics (Average rigid): 3.26392 kg CO_2e per kg<br>- Plastics (PP): 3.09082 kg CO_2e per kg<br>- Plastics (PET): 4.01848 kg CO_2e per kg<br>- Glass: 1.40277 kg CO_2e per kg<br>- Paper: 0.91048 kg CO_2e per kg<br>- Board: 0.8015 kg CO_2e per kg<br><br>The carbon footprint of common sample kit components and example	

			calculations can be found in the detailed guidance.	
8.2. Movement of sample kit materials from manufacturer to CTU<br>8.3. Movement of sample kits from CTU/distributor to participating Sites<br>8.4. Movement of samples from participating sites or patients to central laboratory.	E.g. shipment of blood tubes for sample kits to CTU	Estimated weight of and distance of delivery (t.km):	Please refer to section 1.2. for freight and 4.1. for refrigerated or frozen freight.	
**9. Laboratory**				
9.1. Emissions attributed to lab utilities according to staff FTE	E.g. energy consumption per square metre of laboratory space according to trial staff FTE	Laboratory staff FTE required for the whole trial duration:	**Electricity:**<br>1644.8 kg CO_2e per FTE per year<br><br>Multiply by the laboratory staff FTE required for the whole trial duration.<br><br>**Heating:**<br>1363.2 kg CO_2e per FTE per year<br><br>Multiply by the laboratory staff FTE required for the whole trial duration.	
9.2. Materials/equipment/consumables used in	E.g. centrifuges, refrigerators	kWh usage of equipment:	To avoid double counting, use of equipment will be included in	

processing and analysis of samples		Kg of material:	lab staff FTE if calculated.<br><br>If the trial does not involve a central lab, but there is still sample processing on site, please see below. For storage of samples, please see section 9.3.<br><br>To calculate the emissions of a piece of equipment, multiply the power consumption by hours used to get a kWh value. Finally multiply kWh by the electricity emission factor (0.257).<br><br>Example: use of a 310-Watt centrifuge for 15 minutes<br>- 310 Watts x 0.25 (hours) = 77.5 kWh<br>- 77.5 kWh x 0.257 = 19.9 kg CO_2e<br><br>Consider the centrifuge capacity and multiply by the number of uses required.	
9.3. Storage of samples	E.g. utilities and ultra-low temperature freezer	Duration of storage:<br><br>Amount of refrigerator/freezer space required:	Storage in fridge/-20 freezer:<br>- 281.4 kg CO_2e per year<br>- Multiply by number of years stored	

			Storage in an ultra-low/-80 freezer:<br>- 2063.7 kg CO_2e per year<br>- Multiply by number of years stored<br><br>NB: this is for a whole freezer; you will need to make an assumption about the amount of space in the freezer that the trial samples take up. As a guide, a typical ULT freezer at full capacity will store 50,000 microtubes.	
**10. Trial close out**				
10.1. Storage and archiving of essential trial documentation and data	E.g. Hospital files, lab files, trial guidance documents etc	Duration of storage:<br><br>Amount of space required for storage:	Carbon footprint associated with $1m^2$ for 1 year:<br>- Office: 17.48 kg CO_2e<br>- Laboratory: 41.1 kg CO_2e<br>- Warehouse: 7.45 kg CO_2e<br>- Health building: 22.1 kg CO_2e<br><br>Choose the most suitable building type and multiply by number of years and m^2 necessary.	

			NB: Approximately 12 archive boxes fit inside 1m^2.<br><br>**Heating**<br>Carbon footprint associated with 1 m^2 for 1 year:<br><br>- Office: 36 kg CO_2e<br>- Laboratory: 34.08 kg CO_2e<br>- Warehouse: 13 kg CO_2e<br>- Health building: 40.04 kg CO_2e<br><br>Choose the most suitable building type and multiply by number of years and m^2 necessary.<br><br>**Electronic data and information storage:**<br>estimate 1.285 kg CO_2e per GB per year.	
10.2. Storage and destruction of biological samples	E.g. blood, tissue, urine etc	M^2 required for storage:<br><br>Duration of storage:	See section 9.3. for storage of refrigerated or frozen samples.<br><br>See section 10.1 for storage of ambient samples.	
10.3. Return of equipment and supplies from	E.g. wearables, unused or expired equipment and	Estimated weight and distance of delivery (t.km):	See section 1.2. (freight).	

participating sites to CTU	supplies			

Carbon footprint summary

Module	KgCO_2e
Trial set up	
CTU emissions	
Trial staff meetings and travel	
Treatment intervention	
Data collection and exchange	
Trial supplies and equipment	
Trial specific patient assessments	
Samples	
Laboratory	
Analysis and trial close out	
**Total =**	

References

---

[1]What is an emission factor? [Internet]. Climfoot-project.eu. [cited 2023 May 11]. Available from: https://climfoot-project.eu/en/what-emission-factor

For all emission factor and benchmark data sources, please refer to the accompanying "Detailed Guidance and method to calculate the carbon footprint of a clinical trial."
