# Supplementary material for: What is the carbon footprint of academic clinical trials? A study of hotspots in 10 trials
Source: BMJ Open. 2024 Oct 16;14(10):e088600. doi: 10.1136/bmjopen-2024-088600 (PMC11487931; doi:10.1136/bmjopen-2024-088600)
Supplement: Supplementary file 4 [file bmjopen-2024-088600.reviewer_comments.pdf]

PEER REVIEW HISTORY

BMJ Open publishes all reviews undertaken for accepted manuscripts. Reviewers are asked to complete a checklist review form and are provided with free text boxes to elaborate on their assessment. These free text comments are reproduced below.

ARTICLE DETAILS

**TITLE (PROVISIONAL)**	What is the carbon footprint of academic clinical trials? A study of hotspots in 10 trials
**AUTHORS**	Griffiths, Jessica; Adshead, Fiona; Al-Shahi Salman, Rustam; Anderson, Craig; Bedson, Emma; Bliss, Judith; Boshoff, Ana; Chen, Xiaoying; Cranley, Denise; Doran, Peter; Dunne, Fidelma; Gamble, Carrol; Gillies, Katie; Hood, Kerenza; Kavanagh, Columb; Malone, Julia; McGregor, Naomi; McNamara, Carolyn; Midha, Elis; Moore, Keith; Murphy, Lucy; Newman, Christine; O'Reilly, Seamus; Perkins, Alexis M; Pett, Sarah; Sydes, Matthew; Whitty, Laura; You, Frank; Fox, Lisa; Williamson, Paula

VERSION 1 - REVIEW

**REVIEWER NAME**	***LaRoche, Jason***
**REVIEWER AFFILIATION**	Janssen Research and Development LLC, Janssen Clinical Innovation
**REVIEWER CONFLICT OF INTEREST**	Na
**DATE REVIEW RETURNED**	23-May-2024

**GENERAL COMMENTS**	I appreciate the opportunity to review your manuscript. Your submitted manuscript is well written and does a good job explaining your research in an easily readable format. I have five comments or suggested minor revisions:<br><br>1) In Table-2, why is sustenance included in the analysis? If people will eat lunch and dinner anyways, why count this as something new or additive to the existing ecosystem? I would only include sustenance where the meals may feature a diet that is different from one that might normally be consumed and would have a unique additive environmental impact.<br><br>2) Is it correct that you do not account for emissions from the manufacture of the treatment intervention? Manufacturing is not mentioned in Table-2 implying that it has been excluded. Any inclusion/exclusion should be more clearly stated. If it is excluded, why? Is it a lack of available data? Rationale for exclusion should be included in the limitations section to acknowledge that this important piece is missing.<br><br>3) On page 13, lines 29-36 are repetitive as this same text appears in the main body of the text under the Introduction section. Consider removing.<br><br>4) While I appreciate the simplicity of Table-3 and how it identifies the three major hotspots that contribute to each study's emissions, as a reader I'm left wanting to know the magnitude of the hotspot contributions. Please consider adding the quantified contribution of

	each of the three major hotspots to the overall total.<br><br>5) Page-16, row-17, replace the abbreviation "GB" with the word "gigabyte". The reader may lack sufficient context to quickly associate the abbreviation with the associated unit measure for data storage.<br><br>Attached you will find these same comments highlighted directly in the submitted copy of your manuscript.

**REVIEWER NAME**	***Mackillop, Neil***
**REVIEWER AFFILIATION**	AstraZeneca PLC, Late Development CVRM
**REVIEWER CONFLICT OF INTEREST**	Na
**DATE REVIEW RETURNED**	06-Jun-2024

**GENERAL COMMENTS**	Having reviewed the guidance document in detail for a previous publication I have not re-reviewed. One general comment that is perhaps more for the Journal is whether previous published versions of the Guidance should now be withdrawn so that all future users use the current improved version.<br>At the bottom of my review I have pasted comments from a colleague who independently reviewed all the materials. Although his background in emissions work is different from mine his comments on the main paper are remarkably similar to mine.<br><br>Generally "line number" refers to the actual line on the relevant page ie ignoring the double spacing. In some places it was easier to refer to "marked line" which refers to the editorial marking on the left hand side of the page with numbers ranging from 0-60<br>Abstract<br>para 1 line 4 ? "continue" rather than "begin"<br>Para 2 methods. One important aim that is rather underplayed in the whole paper, is the need to assess the time and skill needed by those completlng these LCAs. In this para it is unclear who is collating and who multiplied...but it is clear in the main article including the sites who needed extra help...and maybe word counts preclude more detail here<br>Para 3 My personal pref is to write in the active eg to start We footprinted ..... which in this case removes any ambiguity what the "variety of interventions" refers to. (and can it be "6" rather than "six"?)<br>S and L<br>First line "triallists who" removes a sl ambiguity regarding who has the "no experience"<br>Bullet 4 remove "by paid subscription" because that wont always be the case<br>Bullet 5 the ending might confuse those not sure what an LCA is. I suggest to end with " ....other env impacts such as water, air quality etc"<br>Introduction<br>Given the ref to 2023 in para 1 line 6, I would take Lyle out as a reference. We know it had many weaknesses and your current data is reflects the current world and is better quality data.<br>End of line 8 (and in many other places in this paper where I wont repeat this comment) I suggest to remove the decimal place which gives an impression of accuracy that doesnt exist with these figures (lets watch UK national figures change if the govt changes!)

	Para 2 Line 1 (Didnt we already pass it Feb 2023-Feb 2024?)<br>Para 2 last line "we developed"...is that in this paper or a reference to your previous pubs? might be clarified by removing para break to the next para<br>Method--Trial Selection<br>Will an ex-UK or non-academic audience wonder what are the organisations mentioned in the first 2 lines<br>Table 1 I and an intern who looked at this paper wondered whether Table 1 should be "demoted" to supplementary material...where those who want to can check the details of "wide ranger of academic trials"<br>Table 2 It would be ideal to clearly explain/justify the basis for selecting the 10 modules and the "content" of each, for example other pubs from pharma have focussed on hotspots of "travel" and "waste" (admittedly a lot of the waste related "drug product) and also Samples" but in the pharma case this has included what you have called "Laboratory"<br>Page 10 Top para last line: I suggest to remove "..whic would be barrier for public funded trialists" because it is a very imperfect mapping<br>Second para It would be ideal to calculate/give idea of sensitivity to use of UK as surrogate for other countries<br>Page 11 Table 3 Can this be combined with Table 1? I wondered about the value of ranked hotspots; the intern wanted the actual figure for the 3 hot spots for each trial but then it duplicates your more detailed data breakdown<br>Pag 13 line 3 Can you give some sense of how reliable/accurate these proxy figures are...or give some examples<br>p13 Total carbon footprint Suggest to remove second para of this section which seems a repeat of a para in the intro<br>Carbon hotspots<br>First para line 3 "longer trial duration" in a rather hidden way this is defining what you mean by hotspot ie it is total emissions over time not intensity of emissions<br>P14 LINE 2 I think it is safe to remove "may"<br>Line 4 Omitting standard of care should be given more prominence. it is an important aspect of methodology<br>pag14 marked as line 40 ULT might be an unknown abbreviation to some readers. How is 10 years storage counted given it probably hasnt completed yet?<br>Para marked as line 43-53 Was single use assumed for devices etc. What happens if bought for our study trial and then put into general use in the clinic?<br>page 15 marked as line 19 I guess here financial cost is being used as a surrogate/proxy but for software that seems to be likely to be a big over-estimate of emissions<br>page 15 Feedback on C calcs A better title could be "Time required to collect the activity data to enable carbon calc There is maybe enough data here but somehow it should have more prominence......for some trialists it will be their most important question, issue, barrier. I assume 10-25 hours refers to 1 FTE. Maybe the FTEs used and perhaps the persons role could be added to Table 1 or 3 (wherever you decide to put it/them)<br>P16 line 2 "data collection" is presumably "for carbon calc" not trial data collection its not obvious to me why more assumptions speed data collection...and/or is it making the calc less reliable?! I realise it is difficult to summarise the work involved but hope you can make clear that difficulty is separate from the relative ease of doing the work<br>p16 marked line 43 "comuting using activity data"...I assume this

	means instead of financial cost but it probably needs explaining<br>p16 bottom line.......is there anyone who wont have an accurate figure for how many hours are in a local FTE?<br>Discussion<br>Para 3 The similarity to Lyle and CRASH is interesting. I have tended to think of these publications as rather limited assessments of old trials. Overall do you think these older pubs were as complete as your work, that the activities were done in a similar way and the conversion to emissions was similar......or is it that various of these factors have cancelled each other out (eg uk energy grid emissions must now be lower) YOu refer to using some relatively old emission factor data....could that be impacting your results?<br>p19 First para of Opportunities, last sentence. Yes, altho that requires that you lump commuting into CTU emissions. Depending on climate, home heating, cooling etc it may increase total emissions<br>P19 last line EV may indeed reduce overall emissions but travel emissions would be reduced further by not travelling at all. The idea of offsetting (ie mention of "net") is unhelpful because it takes us into a whole murky area.<br>p20 2nd para second last word "Finish"...is that the correct word or a typo?<br>Marked line 46: I would say "..consideration should be given"<br>page 22 line 5 I would add that the country emission factors "can be used to choose the countries for an international trial"<br>p25 Next steps First sentence. I hope these sites who thought it too burdensome will think again....it would be interesting to ask now that you can probably estimate the time it would have taken them.<br>COI statemnt: Is there a logic to the ordering of individuals?<br>Contributor and Funding Assuming MRS is not Sydes should MRS also have a 4th letter to match MRSy<br>--<br>Below here comments from Sandeep Prakasam (who will also hold confidential the proposed publication and its contents)<br>Review of: Quantifying the carbon footprint of academic clinical trials: building the evidence base and hotspot identification<br>This manuscript builds on the January 2024 report by authors Griffiths, J., Fox, L., and Williamson, P.R. It reports the carbon footprint assessment made by the eight CTUs, consulted for the for original framework. In addition, the emission assessments of two more CTUs, namely Cancer Trials Ireland (Shamrock trial) and The Centre for Healthcare Randomised Trials (INTERVAL trial) are reported.<br>General comment:<br>• The key highlights of this manuscript are (i) the updated guidance (from V0.4 to V0.5), (ii) hotspot analysis of the ten trials, and (iii) triallists' feedback on carbon calculation. Highlighting these aspects and minimizing repetition from the previous manuscript would enhance reader experience.<br>The following are the specific observations on manuscript for the authors' kind consideration:<br>1. Table 1:<br>o Apart from the Shamrock and INTERVAL trials, the description of the other trials were already reported in the previous study. Consider listing the original eight studies and simply referring to the previous manuscript for the full description.<br>o Potentially merge Table 1 with Table 3 for improved organization<br><br>2. New emission factors and activities added to guidance:<br>o This is a significant section of this manuscript, as it highlights the

	changes from V0.4 V0.5. A comparative table outlining specific changes between V0.4 and V0.5 would benefit the readers.<br><br>3. (minor) Table 3:<br>o I wonder if including the % contribution values next to "1'," "2'," and "3'" hotspots (already shown in Figure 1) would improve table readability.<br><br>4. Discussion, Page 19 Line 56-57: Care should be taken to comment on the emissions from green taxis. Plug-in and hybrid cars have zero to minimum tailpipe emissions, however, the source of electricity generation could contribute to emissions (fossil fuel vs. renewable energy). (https://afdc.energy.gov/vehicles/electric-emissions)<br><br>5. Appendix B:<br>o To reduce redundancy, it may be better to refer to the previous paper and highlight only the changes.<br><br>o The major changes are:<br>i. UK GHG conversion factor (2021 vs. 2023) and Office benchmark and natural gas emissions (2023 vs. 2022)<br>ii. Inclusion of blood pressure monitors<br>iii. Updated benchmark for radiotherapy and other materials (section 7.2)<br><br>o The emissions benchmarks in the UK (and other nations) are likely to be updated every year. Therefore, providing a commentary on evolving benchmarks and where to find the latest values to do the calculations will be beneficial to the reader in the long term. The assumptions used in Jan 2024 manuscript have already been outdated, and the assumptions used in the current manuscript are likely to be dated in a few months.<br>6. Appendix Section 5 and section 10.1:<br>o The original source of the calculation of 5kwh per GB of data comes from this study : https://www.aceee.org/files/proceedings/2012/data/papers/0193-000409.pdf (which was referred in the Medium article). Consider updating the reference to the original source material.<br>o Understandably, this must include a caveat as the report is from 2012. The internet infrastructure, mobile networks and energy efficiencies have improved since then. Further data storage and sharing have increased in complexity.<br>7. The Appendix C:<br>o While similar to the previous manuscript (Supp Matt 2), this appendix can likely stay, as this worksheet has the updated assumptions and will be relevant to the readers.

VERSION 1 – AUTHOR RESPONSE

Comments from Reviewer 1

Comment: I appreciate the opportunity to review your manuscript. Your submitted manuscript is well written and does a good job explaining your research in an easily readable format.

Response: Thank you for your review and positive feedback.

Suggested revision 1: In Table-2, why is sustenance included in the analysis? If people will eat lunch and dinner anyways, why count this as something new or additive to the existing ecosystem? I would only include sustenance where the meals may feature a diet that is different from one that might normally be consumed and would have a unique additive environmental impact.
Response: In our method, guidance is provided to calculate emissions attributed to sustenance in the 'Trial specific meetings and travel' module only. Our rationale for including this is that there is evidence that people eat differently from usual when away and/or at meetings, for example eating in restaurants where the carbon footprint is higher (https://guide.michelin.com/us/en/article/features/zerofoodprint-restaurants-climate-change). This is an assumption that will be explored further in future work.

Suggested revision 2: Is it correct that you do not account for emissions from the manufacture of the treatment intervention? Manufacturing is not mentioned in Table-2 implying that it has been excluded. Any inclusion/exclusion should be more clearly stated. If it is excluded, why? Is it a lack of available data? Rationale for exclusion should be included in the limitations section to acknowledge that this important piece is missing.
Response: The emissions attributed to IMP/intervention manufacture are excluded. This is noted in the 'Next steps' section of the discussion and the assumptions listed at the start of the Guidance document (appendix 2). However, we have also now stated this in Table 2 as suggested for clarity. The reason for the exclusion is that manufacture of an IMP or other intervention is not an activity trialists can control the environmental impact of, as defined in the guidance document.

Suggested revision 3: On page 13, lines 29-36 are repetitive as this same text appears in the main body of the text under the Introduction section. Consider removing.
Response: We agree and have removed the text mentioned as suggested.

Suggested revision 4: While I appreciate the simplicity of Table-3 and how it identifies the three major hotspots that contribute to each study's emissions, as a reader I'm left wanting to know the magnitude of the hotspot contributions. Please consider adding the quantified contribution of each of the three major hotspots to the overall total.
Response: We agree with this suggestion and have added the % contribution of each ranked hotspot to the total footprint in Table 3. The actual value for each hotspot is available in Figure 1.

Suggested revision 5: Page-16, row-17, replace the abbreviation "GB" with the word "gigabyte". The reader may lack sufficient context to quickly associate the abbreviation with the associated unit measure for data storage.
Response: We agree and have expanded the abbreviation.

Comments from Reviewer 2

Abstract:
Comment 1: para 1 line 4 – suggest "continue" rather than "begin"
Response: We agree and have amended.

Comment 2: Para 2 (methods) - One important aim that is rather underplayed in the whole paper, is the need to assess the time and skill needed by those completing these LCAs. In this para it is unclear who is collating and who multiplied, but it is clear in the main article

including the sites who needed extra help.
Response: We agree that this is a good point to highlight in the abstract and have amended the final sentence of paragraph 2 to include mention of this.

Comment 3: Para 3 - My personal preference is to write in the active e.g. to start “We footprinted” which in this case removes any ambiguity what the "variety of interventions" refers to (and can it be "6" rather than "six"?).
Response: We have amended the paragraph to start with “We footprinted”. We have left as “six” because throughout the manuscript we have followed the standard practice of writing out numbers below 10.

Strengths and Limitations section:
Comment 4: First line - "trialists who" removes a strengths/limitations ambiguity regarding who has the "no experience".
Response: Changed “trialists with” to “trialists who have” as suggested.

Comment 5: Bullet 4 - remove "by paid subscription" because that won’t always be the case
Response: We have inserted the word “often” before the text mentioned to account for this not always being the case.

Comment 6: Bullet 5 - the ending might confuse those not sure what an LCA is. I suggest to end with " ....other env impacts such as water, air quality etc".
Response: We agree and have revised as suggested.

Introduction:
Comment 7: Given the ref to 2023 in para 1 line 6, I would take Lyle out as a reference. We know it had many weaknesses and your current data is reflects the current world and is better quality data.
Response: We have referred to the Lyle work because we feel it is important to reference our work within the context of other academic trial footprinting work.

Comment 8: End of line 8 (and in many other places in this paper where I won’t repeat this comment) I suggest to remove the decimal place which gives an impression of accuracy that doesn’t exist with these figures.
Response: We agree and have removed decimal places as suggested throughout the manuscript.

Comment 9: Para 2 Line 1 - Didn’t we already pass the 1.5 degree threshold Feb 2023-Feb 2024?
Response: The 1.5 target set in the Paris agreement is the target average temp over decades, but we agree this sentence should be clearer. We have updated paragraph 2 line 1 of the introduction to clarify.

Comment 10: Para 2 last line "we developed"...is that in this paper or a reference to your previous pubs? Might be clarified by removing para break to the next para.
Response: That is in reference to our previous publication. We agree and have removed the paragraph break as suggested.

Method - Trial Selection
Comment 11: Will an ex-UK or non-academic audience wonder what are the organisations mentioned in the first 2 lines.
Response: We agree with this point and have therefore added references for the

organisations.

Comment 12: Table 1 - I and an intern who looked at this paper wondered whether Table 1 should be "demoted" to supplementary material...where those who want to can check the details of "wide ranger of academic trials"
Response: We feel that it is important that table 1 remains in the main manuscript document as understanding the nature of each trial is crucial to understanding the results in Table 3.

Method – Calculation of trial carbon footprint:
Comment 13: Table 2 - It would be ideal to clearly explain/justify the basis for selecting the 10 modules and the "content" of each, for example other pubs from pharma have focussed on hotspots of "travel" and "waste" (admittedly a lot of the waste related "drug product) and also Samples" but in the pharma case this has included what you have called "Laboratory".
Response: The content of each module is explained in Table 2 and the modular approach was explained in our previous BMJ Open publication about development of the method. However, we agree that the basis for the modular structure should also be explained here so we have added additional wording to page 9 line 24 to clarify (above table 2) - "The content and structure of the modules reflect the funding, governance and trial management structures of academically-funded clinical trials"

Comment 14: Page 10 Top para last line - I suggest to remove "..which would be barrier for public funded trialists" because it is a very imperfect mapping.
Response: We believe this would often be this case however understand that there could be instances where it is not, so we have changed the wording from 'would' to 'could'.

Comment 15: Page 10 Second para - It would be ideal to calculate/give idea of sensitivity to use of UK as surrogate for other countries.
Response: It is not possible to perform this sensitivity analysis currently, as UK data is only used where the equivalent country-specific data does not exist/is not publicly available. We have added examples of where country specific data was or wasn't used.

Results
Comment 16: Page 11 Table 3 - Can this be combined with Table 1? I wondered about the value of ranked hotspots; the intern wanted the actual figure for the 3 hot spots for each trial but then it duplicates your more detailed data breakdown.
Response: We have added the percentage contribution of each ranked hotspot to Table 3. The actual figure for the hotspots are available in figure 1.

Comment 17: Pag 13 line 3 - can you give some sense of how reliable/accurate these proxy figures are...or give some examples.
Response: We have removed this sentence because it was in reference to international emission factors which has been explained in the method section and now expanded in response to comment 15. We were able to identify emission factors to carbon footprint all other trial activities.

Comment 18: p13 Results - Total carbon footprint section - Suggest removing second para of this section which seems a repeat of a para in the intro.
Response: We agree and have removed as suggested.

Results - Carbon hotspots:
Comment 19: First para line 3 - "longer trial duration" in a rather hidden way this is defining what you mean by hotspot i.e. it is total emissions over time not intensity of emissions?

Response: We are not making a point about intensity vs duration, we are stating the observation that CTU emissions tend to be a hotspot in longer duration trials. We have amended the end of the sentence to clarify.

Comment 20: P14 line 2 - I think it is safe to re/move "may".
Response: We agree and have removed.

Comment 21: Line 4 - Omitting standard of care should be given more prominence, it is an important aspect of methodology.
Response: We have stated this in the method section and in the scope of the guidance document (appendix 2). However, we have added mention of this to Table 2 to clarify.

Comment 22: page 14 marked as line 40 - ULT might be an unknown abbreviation to some readers. How is 10 years storage counted given it probably hasn't completed yet?
Response: We have expanded the abbreviation as suggested. The footprint of the trial includes the footprint of planned and funded activities which includes the planned storage of samples for a defined period of time, therefore where known, this was counted.

Comment 23: Para marked as line 43-53 - Was single use assumed for devices etc. What happens if bought for our study trial and then put into general use in the clinic?
Response: Single use was assumed if the trial duration (years) and the estimated device usage (hours) during the trial reached the assumed life span of the device. However, within the guidance, instructions for calculating the emissions attributed trial usage only are included.

Comment 24: page 15 marked as line 19 - I guess here financial cost is being used as a surrogate/proxy but for software that seems to be likely to be a big over-estimate of emissions.
Response: Yes, financial cost is used for software and we agree this is likely a large over-estimate of emissions. However, at this stage we are unable to get a better activity-based estimate, and this is acknowledged in the next sentence of that paragraph – "However, in the absence of sufficient activity data and published emission factors, emissions attributed to these activities were calculated using a spend-based emission factor, which are known to be less accurate than activity-based emission factors. "

Comment 25: page 15 Feedback on C calcs - A better title could be "Time required to collect the activity data to enable carbon calc". There is maybe enough data here but somehow it should have more prominence......for some trialists it will be their most important question, issue, barrier. I assume 10-25 hours refers to 1 FTE. Maybe the FTEs used and perhaps the persons role could be added to Table 1 or 3 (wherever you decide to put it/them)
Response: We have amended the title to "Feedback on application, use and experience of carbon footprinting guidance". We did not have complete enough data to add time feedback to Table 3.

Comment 26: P16 line 2 - "data collection" is presumably "for carbon calc" not trial data collection. Its not obvious to me why more assumptions speed data collection...and/or is it making the calc less reliable?! I realise it is difficult to summarise the work involved but hope you can make clear that difficulty is separate from the relative ease of doing the work.
Response: In the limitations section of the discussion we acknowledge that the footprint of a trial calculated prospectively may differ from that of the completed trial and that the intention of doing it prospectively is not to calculate an absolute footprint, but to enable lower carbon trials by comparison of alternative design options. Calculating prospectively allows the user to use existing assumptions that have already been made to inform an academic trial funding application, and as such collation of activity data is more straight forward than searching

records for a completed trial for the absolute quantity of a particular activity. We have amended page 16 line 2 to clarify this.

Comment 27: p16 marked line 43 - "commuting using activity data"...I assume this means instead of financial cost but it probably needs explaining.
Response: Previously we only provided an average commuting emission factor. I have amended the sentence to clarify.

Comment 28: p16 bottom line - is there anyone who won't have an accurate figure for how many hours are in a local FTE?
Response: We have provided an assumption for the average number of working hours in a year because this will depend on the amount of leave and sick days taken. Where the person conducting the carbon calculations is doing so on behalf of a trial team, they may not be able to acquire this information. The total number of working hours is required to calculate emissions attributed to working from home.

Discussion:
Comment 29: Para 3 - The similarity to Lyle and CRASH is interesting. I have tended to think of these publications as rather limited assessments of old trials. Overall do you think these older pubs were as complete as your work, that the activities were done in a similar way and the conversion to emissions was similar......or is it that various of these factors have cancelled each other out (eg uk energy grid emissions must now be lower) You refer to using some relatively old emission factor data....could that be impacting your results?
Response: We agree that the application of the methodology in the CRASH-1 and CRASH-2 trials was slightly different and have added acknowledgement of this to the text. However, we feel it is important to reference our work within the context of other academic trial footprinting work.

Comment 30: p19 First para of Opportunities, last sentence (Hybrid working will also contribute to reduced CTU emissions due to reduced commuting). Yes, altho that requires that you lump commuting into CTU emissions. Depending on climate, home heating, cooling etc it may increase total emissions.
Response: Commuting is included in our CTU emissions calculations. When commuting is taken into account it is very unlikely that working from home or hybrid working would have higher emissions than 100% office based, even with increased emissions from heating and cooling.

Comment 31: P19 last line EV may indeed reduce overall emissions but travel emissions would be reduced further by not travelling at all. The idea of offsetting (ie mention of "net") is unhelpful because it takes us into a whole murky area.
Response: We agree that not travelling at all would have the biggest impact on reducing emissions from travel. However, travel will be necessary in some trials and where it is, travel via electric vehicles would reduce emissions. We agree mention of net zero can be unhelpful and therefore it has been removed.

Comment 32: p20 2nd para second last word "Finish"...is that the correct word or a typo?
Response: That is the correct word, it is the name of the group.

Comment 33: Marked line 46: I would say "..consideration should be given".
Response: We agree and have amended as suggested.

Comment 34: page 22 line 5 I would add that the country emission factors "can be used to

choose the countries for an international trial"
Response: Due to other considerations and trade-offs we wouldn’t be choosing countries for an international trial based only on emissions, however we have added wording to the sentence referenced to say that we’d also like to “understand country-specific trial emissions”.

Comment 35: p25 Next steps First sentence. I hope these sites who thought it too burdensome will think again....it would be interesting to ask now that you can probably estimate the time it would have taken them.
Response: Good point and we’ll consider that going forward.

Comment 36: COI statement: Is there a logic to the ordering of individuals?
Response: We have amended so the ordering is alphabetical.

Comment 37: Contributor and Funding - Assuming MRS is not Sydes should MRS also have a 4th letter to match MRSy.
Response: Yes, we have amended.

Comments from Sandeep Prakasam
The following are the specific observations on manuscript for the authors’ kind consideration:
1. Table 1:
o Apart from the Shamrock and INTERVAL trials, the description of the other trials were already reported in the previous study. Consider listing the original eight studies and simply referring to the previous manuscript for the full description.
Response: We have been advised by the editor not to do this - “As this is a stand-alone publication, we suggest to keep the description of the included trials instead of just referencing the previous publication for eight of the included trials as suggested by Sandeep Prakasam. Similarly regarding Appendix B.”

o Potentially merge Table 1 with Table 3 for improved organization.
Response: We felt that the information captured in Table 1 is an introduction to the trials and therefore critical to understanding the results in Table 3.

2. New emission factors and activities added to guidance:
o This is a significant section of this manuscript, as it highlights the changes from V0.4 V0.5. A comparative table outlining specific changes between V0.4 and V0.5 would benefit the readers.
Response: All changes between v0.4 and v0.5 have been stated in the results section. We have also updated appendix 2 to include a table of changes in the guidance itself.

3. (minor) Table 3:
o I wonder if including the % contribution values next to "1'," "2'," and "3'" hotspots (already shown in Figure 1) would improve table readability.
Response: We agree with this suggestion and have amended Table 3 to include this.

4. Discussion, Page 19 Line 56-57: Care should be taken to comment on the emissions from green taxis. Plug-in and hybrid cars have zero to minimum tailpipe emissions, however, the source of electricity generation could contribute to emissions (fossil fuel vs. renewable energy). (https://afdc.energy.gov/vehicles/electric-emissions)
Response: We agree and have amended these lines to instead focus on and advocate for renewable energy powered EV’s.

5. Appendix B:

o To reduce redundancy, it may be better to refer to the previous paper and highlight only the changes.
Response: Editor advised against this, stating "As this is a stand-alone publication, we suggest to keep the description of the included trials instead of just referencing the previous publication for eight of the included trials as suggested by Sandeep Prakasam. Similarly regarding Appendix B".

o The major changes are:
i. UK GHG conversion factor (2021 vs. 2023) and Office benchmark and natural gas emissions (2023 vs. 2022)
ii. Inclusion of blood pressure monitors
iii. Updated benchmark for radiotherapy and other materials (section 7.2)
o The emissions benchmarks in the UK (and other nations) are likely to be updated every year. Therefore, providing a commentary on evolving benchmarks and where to find the latest values to do the calculations will be beneficial to the reader in the long term. The assumptions used in Jan 2024 manuscript have already been outdated, and the assumptions used in the current manuscript are likely to be dated in a few months.
Response: We agree and have added wording to the discussion – "The guidance will also be updated in line with evolving emission factors and future iterations will be published on the TMRP website."

6. Appendix Section 5 and section 10.1:
o The original source of the calculation of 5kwh per GB of data comes from this study : https://www.aceee.org/files/proceedings/2012/data/papers/0193-000409.pdf (which was referred in the Medium article). Consider updating the reference to the original source material.
o Understandably, this must include a caveat as the report is from 2012. The internet infrastructure, mobile networks and energy efficiencies have improved since then. Further data storage and sharing have increased in complexity.
Response: Thank you for providing the original reference, we have now updated in appendix B. We plan to source a more up to date emission factor in the next iteration of the guidance.

VERSION 2 – REVIEW

**REVIEWER NAME**	***LaRoche, Jason***
**REVIEWER AFFILIATION**	Janssen Research and Development LLC, Janssen Clinical Innovation
**REVIEWER CONFLICT OF INTEREST**	With some of the authors, I am involved in research and discussions separate but tangential to the research enclosed in this manuscript.
**DATE REVIEW RETURNED**	12-Aug-2024

**GENERAL COMMENTS**	Thank you for submitting amendments to the original manuscript and considering my previous suggestions. I was happy to see that you provided additional clarity around the exclusion of the environmental footprint of investigational products, as this was not clear in the previous manuscript. You also included the percentage contribution in additional to the ordinal ranking of the hotspots, which provides the reader with a better sense of the relative impact on the overall study emissions. From my point of view, the only remaining gap is a rationalization for why the environmental impact of sustenance for patients was included. One would argue that

	patients will eat anyways, so if a patient is already consuming meals then is the trial really influencing a change from normal routine? My rationale for inclusion would be if your are carefully controlling diet in a way that would cause meals to be different from those normally consumed. Even in that case, I would attribute only the difference between the environmental impact of the provided meal and a patient's normal meal routine in your analysis. This gap does not diminish the quality of this manuscript and I feel that the manuscript is worthy of publication, but the inclusion of sustenance will likely draw questions and scrutiny from readers well versed in LCA methodology.

**REVIEWER NAME**	***Mackillop, Neil***
**REVIEWER AFFILIATION**	AstraZeneca PLC, Late Development CVRM
**REVIEWER CONFLICT OF INTEREST**	Na
**DATE REVIEW RETURNED**	09-Aug-2024

**GENERAL COMMENTS**	Thanks for the very complete and detailed responses to the reviewer comments that have allowed a rapid re-review. I hope this paper can now rapidly go to publication because there is a need and demand from many stakeholders for this data.

VERSION 2 – AUTHOR RESPONSE

Reviewer 1 comment: From my point of view, the only remaining gap is a rationalization for why the environmental impact of sustenance for patients was included. One would argue that patients will eat anyways, so if a patient is already consuming meals then is the trial really influencing a change from normal routine? My rationale for inclusion would be if you are carefully controlling diet in a way that would cause meals to be different from those normally consumed. Even in that case, I would attribute only the difference between the environmental impact of the provided meal and a patient's normal meal routine in your analysis. This gap does not diminish the quality of this manuscript and I feel that the manuscript is worthy of publication, but the inclusion of sustenance will likely draw questions and scrutiny from readers well versed in LCA methodology.

Response: Sustenance for patients was not included in the carbon footprint of the trials. The carbon footprint of sustenance is only calculated in the ‘Trial specific meetings and travel’ module. This includes catering provided at trial meetings and/or sustenance consumed whilst staff travel on trial specific business. Our reason for including this is that there is evidence that people eat differently from usual when travelling for business and/or at meetings, for example eating in restaurants where the carbon footprint is higher (https://guide.michelin.com/us/en/article/features/zerofoodprint-restaurants-climate-change). This is an assumption that will be explored further in future work and we have added mention of this to the discussion.
